# Addition of CAD polygenic risk score to coronary artery calcium score enhances prediction of MACE

**DOI:** 10.3389/fcvm.2026.1814510

**Published:** 2026-06-04

**Authors:** Andrii Maryniak, Filip Oleszak, Kevin Coy, Eric Larson, Catherine Hajek, Shubhi Bartaria, Shaopeng Gu, Max Weaver, Jerome I. Rotter, Xiuqing Guo, Jingyi Tan, Henry J. Lin, Adam Stys, Natalia Baran, Tomasz Stys

**Affiliations:** 1 Sanford Cardiovascular Institute, University of South Dakota Sanford School of Medicine, Sioux Falls, SD, United States; 2 Sanford Health, Sioux Falls, SD, United States; 3 Helix LLC, San Mateo, California, United States; 4 Institute for Translational Genomics and Population Sciences, Department of Pediatrics, The Lundquist Institute for Biomedical Innovation at Harbor-UCLA Medical Center, Torrance, CA, United States; 5 Department of Hematology and Hematological Central Laboratory, InselSpital Bern, Bern University Hospital, Bern, Switzerland; 6 Department of Internal Medicine, University of South Dakota Sanford School of Medicine, Sioux Falls, SD, United States, United States

**Keywords:** cardiac genetics, cardiovascular risk prediction, coronary artery calcium score, polygenic risk score, primary prevention

## Abstract

**Background:**

Coronary heart disease (CHD) is prevalent in the United States, highlighting the need for accurate risk prediction to inform primary prevention strategies. While multivariate risk models like the Framingham Risk Score and ACC/AHA Pooled Cohort Equations are commonly utilized, novel risk markers, such as the coronary artery calcium score (CACS) and polygenic risk score (PRS), are increasingly gaining recognition.

**Objectives:**

This study aimed to compare the diagnostic utility of CACS and CAD PRS, both individually and in combination, for predicting major adverse cardiovascular events (MACE).

**Methods:**

We conducted a retrospective analysis of a cohort comprising 1,380 predominantly Caucasian participants from the Sanford Health System. CAD PRS was constructed using genetic variants, while CACS was assessed via cardiac computed tomography (CT). Statistical analyses evaluated the relationship between each modality and MACE.

**Results:**

Both CAD PRS and CACS were significantly associated with future MACE. Following the adjustment for covariates, the area under the curve (AUC) for both the CACS and PRS models was comparable, indicating similar predictive capabilities for MACE. However, the combination of CAD PRS with CACS significantly enhanced predictive accuracy, outperforming either modality alone.

**Conclusions:**

This study underscores the value of integrating CACS and CAD PRS in predicting MACE. The synergistic effect of CAD PRS combined with CACS markedly improves predictive power. Further research and prospective studies are necessary to validate these findings and assess their clinical implications. Investigating the interactions between PRS and CACS will be crucial for refining cardiovascular risk prediction and optimizing prevention strategies.

## Introduction

In the United States, coronary heart disease (CHD) affects approximately 7.2% of adults aged 20 years and older (1). Accurate risk prediction is essential for the primary prevention of cardiovascular disease (CVD). Estimating an individual's absolute risk of CVD relies on multivariate risk models, including the Framingham Risk Score (2), the American College of Cardiology/American Heart Association (ACC/AHA) Pooled Cohort Equations (PCEs) (3), QRISK3 (4), and European Society of Cardiology SCORE2 model (5).

Alongside these guideline-recommended risk models, novel risk markers such as the coronary artery calcium score (CACS) and polygenic risk score (PRS) are redefining cardiovascular risk assessment. CACS, which quantifies the amount of calcium in the coronary arteries, determined through computed tomography (CT), predicts future coronary events by assessing the extent of subclinical atherosclerosis, and thus serves as an indicator of atherosclerotic burden (6). Higher CAC scores are associated with an increased risk of coronary artery disease and cardiovascular events. Moreover, for patients identified as low risk for coronary artery disease (CAD) based on traditional cardiovascular risk factors, CACS enhances the estimation of obstructive CAD risk, aiding in the reclassification of individuals into higher or lower risk categories and informing preventative strategies, including statin prescription (7–9).

Furthermore, recent advancements in genome-wide association studies (GWAS) have facilitated the construction of polygenic risk scores (PRSs), derived from genetic variants associated with coronary artery disease (10). PRS combines the effects of multiple genetic loci to provide an estimate of an individual's genetic predisposition to developing CAD, thus representing the cumulative risk conferred by multiple disease-associated single nucleotide variants (11). Genomic screening, which can be performed early in life (12), complements traditional risk prediction methods (10) and identifies high-risk individuals before the onset of comorbid conditions (13). The clinical utility of incorporating PRS and CACS into conventional risk assessment models has been validated individually: CAC scores can delineate the risk based on existing atherosclerotic disease, while PRS facilitates earlier prediction of cardiovascular events independently of established clinical and demographic risk factors (11–15).

Similarly, CACS has been strongly associated with the 10-year risk of incident CHD in a graded manner, providing a more accurate estimation of the pretest probability of obstructive coronary artery disease (CAD). This association is particularly beneficial in younger populations, facilitating informed discussions about primary prevention (6, 7, 16).

Major adverse cardiovascular events (MACE), defined as the 4-point composite of acute myocardial infarction, stroke, coronary revascularization, and cardiovascular death, represent the most clinically relevant outcomes for cardiovascular risk prediction models. Therefore, combining these two risk assessment tools may improve the stratification of patients at risk for MACE, allowing for more personalized preventative strategies and treatment interventions. Given the demonstrated success of these novel risk factors, we defined two primary objectives in this study. First, to compare the diagnostic utility of CACS and PRS, both individually and in combination, to enhance the prediction of future MACE, and to evaluate whether the addition of a genome-wide polygenic risk score for coronary artery disease to coronary artery calcium scoring improves prediction of MACE in a large clinical biobank cohort. Second, to validate these findings in two independent external cohorts (the Rotterdam Study and the Multi-Ethnic Study of Atherosclerosis) to assess generalizability across different populations and geographic settings.

In patients with established atherosclerotic disease or multiple risk factors managed in routine practice, MACE is observed in 1.4% of all patients after 1-year and 6.9% at 4-years. In comparison, 1-year incidence was 2.1% in patients with existing CAD and 1.1% in those known to have a high-risk factor burden (17). We hypothesized that the combination of CAC score and PRS would provide superior risk discrimination compared to either measure alone, particularly in individuals with zero or low CAC scores where traditional imaging provides limited risk stratification.

## Methods

### Study population

The study cohort comprised 1,380 participants from the Sanford Health System enrolled between 2011 and 2021, who participated in both the Sanford Heart Screening and the Imagenetics or Biobank programs. All participants underwent CACS measurement and genotyping. The parent population includes 26,291 predominantly Caucasian individuals from North Dakota, South Dakota, Minnesota, and Nebraska. The biobank cohort consists of individuals aged 18 years and older within the Sanford Health system who voluntarily provided specimens for research purposes. The Imagenetics cohort includes participants who enrolled in a preemptive genetic screening program. Data for both cohorts were collected under an exempt study protocol. As the investigators could not readily identify the subjects, the Sanford Health Institutional Review Board (IRB) granted waivers for HIPAA authorization and informed consent.

The Sanford Heart Screening program provides comprehensive preventative heart and vascular health assessments, including blood pressure measurements, body mass index calculations, cholesterol testing, CT Calcium scoring, electrocardiograms (EKG), and Framingham risk scoring. This cohort was selected for its clinically-integrated biobank infrastructure with comprehensive electronic health record linkage enabling longitudinal outcome ascertainment over a median follow-up of 10 years, routine clinical CAC scoring, providing real-world clinical data rather than research-specific imaging, available genetic data, enabling PRS construction; and geographically and socioeconomically diverse Upper Midwest United States population spanning rural and urban communities.

As participants underwent clinically indicated CAC scoring rather than research-specific imaging, the cohort may be enriched for cardiovascular risk factors compared to population-based cohorts, which should be considered when interpreting absolute risk estimates.

### CAD PRS construction & analysis

#### Case definition

Controls and prevalent cases of coronary artery disease (CAD) were identified using ICD-10 codes from the linked electronic medical record via a retrospective analysis. The ICD-10 codes utilized for case classification included those for myocardial infarction; cardiac complications following myocardial infarction; old myocardial infarction; post-infarction angina; and angina associated with atherosclerotic disease of the coronary arteries. Participants with any of these ICD-10 codes were classified as cases. Sanford Health controls had no ICD-10 codes for the following conditions: atrial fibrillation; angina; cardiac arrest; congestive heart failure; myocardial infarction; peripheral vascular disease; stroke; or transient ischemic attack. Data on traditional CAD risk factors for Sanford Health patients included age, sex, race, weight, height, body mass index, total cholesterol levels, high-density lipoprotein (HDL) cholesterol levels, systolic blood pressure, and history of smoking and diabetes.

To ensure analysis of incident (new-onset) MACE rather than prevalent events, we implemented the following approach: participants with documented history of myocardial infarction, stroke, coronary revascularization, or cardiovascular death prior to baseline CAC imaging were excluded; further a 90-day period after CAC imaging was applied to exclude events that may have been prevalent but not yet documented at baseline; MACE events were defined as those occurring >90 days after baseline CAC imaging; a follow-up time was calculated from 90 days post-CAC imaging to first MACE event, death from any cause, loss to follow-up, or end of study period (provide the final date of study), whichever occurred first. Sensitivity analyses were conducted separately in primary prevention (no prior CAD or revascularization) and secondary prevention (prior CAD but no prior MACE) cohorts.

#### Genetic variant selection

The CAD restricted polygenic risk scores (PRS) contains 181 independent SNPs (PRS 181) selected from 259 genome-wide significant SNPs identified in 12 studies. The base GWAS studies from which these variants and effect sizes were derived were predominantly conducted in individuals of European ancestry (Table 1), consistent with the composition of our discovery cohort (96% Caucasian). We performed several harmonization steps on the identified SNPs, including: converting all SNPs to the same genome build; aligning effect alleles; and omitting duplicate or overlapping SNPs. Details are provided in Supplementary Material. We removed 78 SNPs from the original 259 by LD pruning (r2 < 0.2). Proxies replaced palindromic SNPs with allele frequencies between 0.35 and 0.65 with r2 > 0.8 and D’>0.8. All 181 variants were available in imputed data (with imputation quality r2 > 0.3), and PRSs were calculated using the 181 SNPs (Supplementary Material). Patients were classified into 2 groups based on CAD PRS: High PRS (>=85th percentile) or low/intermediate PRS (< 85th percentile).

**Table 1:** Studies referenced for construction of CAD polygenic risk score.

Study	CAD/controls	CAD-associated loci	Independent variants	Genome-wide significant lead SNPs	Candidate genes	Ref.
van der Harst, P., & Verweij, N.,	60,801/ 123,504	64	70	64	several	(36)
Coronary Artery Disease (C4D) Genetics Consortium	15,420/15,062	5	5	5	LIPA, PDGFD, ADAMTS7-MORF4L1, 7q22 locus, KIAA1462	(37)
Nikpay, M., et al.,	60,801/123,504	95	101	95	several	(38)
Howson, J.M.M., et al.,	60,801/123,504	15	18	15	several	(39)
Klarin, D., et al.	22,233/64,762	11	14	11	several	(40)
Kral, B.G., et al.,	2,146/2,402	1	1	1	CDKN2B	(41)
Nelson, C.P.,	60,801/123,504	11	12	11	several	(42)
Reilly, M.P., et al.,	3,404/3,900	2	N/A	N/A	ADAMTS7, ABO	(43)
Schunkert, H., et al.,	22,233/64,762	13	13	13	N/A	(44)
IBC 50K CAD Consortium	15,596/34,992	160	204	22	49	(45)
Helgadottir, A., et al.,	4,587/12,767	157	205	22	50	(46)
CARDIoGRAMplusC4D Consortium	63,746/130,681	46	104	46	233	(47)

#### Score calculation

Participants were genotyped using the Illumina Global Screening Array version 1. Quality control (QC) of samples at Sanford Health was conducted through the Sanford CHIP Clinical Bioinformatics Pipeline. This pipeline verifies critical parameters, including Sample ID, Estimated Gender, and Sample Plate Name, against records in the Laboratory Information Management System (LIMS) to prevent sample mix-ups. In cases of potential discrepancies or suboptimal quality metrics, additional header information-such as Sample Well, Cluster File, SNP Manifest, and Scanner Data- was generated for detailed scrutiny. QC metrics, including Contamination, Raw Control X, Raw Control Y, Log R Deviation, GC 10, GC 50, Intensity Percentiles X, and Intensity Percentiles Y, were computed to determine whether samples met QC standards.

For both clinical and biobank samples, QC analysis was based on Sample ID, sex, and Call Rate. The Sanford Medical Genetics Laboratory (SMGL) cross-verified IDs and gender against internal records. A call rate threshold of 0.99 was applied for clinical samples, while a threshold of 0.75 was used for biobank samples. Samples passing these criteria proceeded to downstream analyses.

The weighted polygenic risk score (PRS) for coronary artery disease (CAD) was calculated by multiplying the allele count (dosage: 0, 1, or 2) for each “risk” SNP by its beta value (effect size) reported in relevant GWAS. The products of the allele counts and their respective beta values were summed across all associated loci to produce a total score. This score was then scaled by dividing it by the total beta value to ensure consistency. For each individual, the PRS was calculated as the weighted sum of risk alleles: PRS = *Σ*(*β*ᵢ × Gᵢ), where *β*ᵢ is the natural log of the odds ratio for SNP i from the base GWAS, and Gᵢ is the allele dosage (0, 1, or 2) for that individual.

The final PRS score was cross-validated in two additional cohorts: The Rotterdam Study and The Multi-Ethnic Study of Atherosclerosis (18)**.** In the Rotterdam Study, the PRS was significantly associated with incident CAD across all three subcohorts (RS-I: HR 1.22, 95% CI 1.14–1.30; RS-II: HR 1.22, 95% CI 1.05–1.41; RS-III: HR 1.36, 95% CI 1.13–1.65; all *p* < 0.01 per standard deviation of the score). In MESA, the PRS was similarly associated with incident CAD (HR 1.15, 95% CI 1.03–1.29, *p* = 0.017 per SD). When examining high-risk individuals, those in the top 2% of the PRS distribution had substantially higher incident CAD risk compared to the bottom 50% across all Rotterdam subcohorts (RS-I: HR 2.17, 95% CI 1.46–3.24; RS-II: HR 2.30, 95% CI 0.99–5.33; RS-III: HR 4.28, 95% CI 2.02–9.05) and in MESA (HR 2.62, 95% CI 1.51–4.54). The RS-II estimate, while directionally consistent, did not reach statistical significance (*p* = 0.052), likely reflecting reduced statistical power in that subcohort (*n* = 2,151).

Further information regarding SNP selection, weighting, and PRS score calculation can be found in Supplementary Material. Raw PRS values were standardized separately within the European-ancestry subset of each cohort (discovery and validation) by subtracting the cohort-specific mean and dividing by the standard deviation, yielding a distribution with mean=0 and SD = 1. This standardization facilitates interpretation (effect per 1-SD increase in PRS) and comparability across cohorts. For non-European ancestry participants, PRS values were standardized using the European-ancestry mean and SD from the same cohort.

#### CACS protocol

Using a Revolution Apex 256-slice cardiac-gated computed tomography scanner (GE Healthcare, Chicago, Illinois), we performed scans at a rotational speed of 0.28 s, with a slice thickness of 2.5 mm, smart coverage, and output ranging from 50 to 430 mA. Coronary artery calcium was detected, and the CACS calculated by the interpreting cardiologist using the Agatston method. Total CACS values were categorized into three groups: 0–100 (low CACS), 100–300 (moderate CACS), and >300 (high CACS). Low and intermediate CACS values were combined into a single low/intermediate category, representing patients with a CACS =<300, while the high category consisted of patients with a CACS >300.

#### Statistical analysis

Statistical analyses were performed using Fisher's exact test, Pearson's Chi-squared test, and Wilcoxon rank-sum test to evaluate differences between stratified risk classifications (PRS, CACS) and prevalence of MACE. Statistical significance was determined based on *p*-values. Utility of CAD PRS and CACS was assessed in two parts. 1) Stratified associations of risk scores and MACE: we analyzed whether patients with higher CAD PRS or CACS were more likely to experience a future MACE, defined as acute myocardial infarction, stroke, coronary revascularization, or cardiovascular death. Chi-squared tests were employed to evaluate the association of stratified representations of CACS (low: < 100, moderate: 100–300, high: >= 300) and CAD PRS (low: <15th percentile, moderate: 15–85th percentile, high: >= 85th percentile) for future MACE. Percentile cutoffs were selected based on the observed risk gradient across the PRS distribution. An inflection point was observed near the 90th percentile where the odds ratio for MACE rose more steeply and approached 2x. As such we defined >85th percentile as high risk to improve sensitivity, 15–85th percentile as an intermediate-risk group with more gradual risk increase, and <15th percentile as low risk. *Post-hoc* Fisher's exact tests further examined whether individuals in the “high” category for each score were at an increased risk for MACE compared to those in the “moderate/low” categories. 2) Combined Predictive Value of CAD PRS and CACS: to demonstrate the predictive value of combining CAD PRS and CACS for future MACE, we employed multiple logistic regression models. These models assessed the risk associated with the presence of both high CAD PRS and high CACS controlling for relevant covariates. Receiver Operating Characteristic (ROC) curves were generated to compare the predictive strengths of CAD PRS and CACS individually and in combination for future MACE occurrences. 3) Hazard survival amongst stratified risk categories: visualize the time-to-MACE via all combinations of risk score categories (high PRS, high CACS; high PRS, low CACS; low PRS, high CACS; low PRS, low CACS). We used 90 days after the date of heart screening as the index time (t₀). Time-to-MACE was defined as time, in years, from this index point to the first MACE event.

## Results

### Demographic and clinical characteristics of patients with major adverse cardiovascular events suggest significant associations between MACE and various risk factors

First, we aimed to investigate the demographic and clinical characteristics of patients with and without MACE. Retrospectively, within a cohort of 1,380 patients analyzed, there were *N* = 91 who experienced MACE and *N* = 1,289, who did not. The cohort analyzed for demographics and baseline coronary artery disease (CAD) risk factors, was predominantly Caucasian (96%), with the mean age of 61 years, and 64% female participants. Genetic ancestry was determined using principal component analysis of genome-wide SNP data with reference to 1000 Genomes Project populations. The ‘other/unknown’ ancestry category (4%, total *n* = 58) comprised individuals who self-identified as mixed ancestry, or declined to provide ancestry information. Sensitivity analyses were conducted both including and excluding this group, with materially unchanged results (*Δ*C-index=0.748 vs. 0.740 in the main analysis). The prevalence of diabetes mellitus, hyperlipidemia, and systolic blood pressure exceeding 120 mmHg was 8.3%, 31%, and 37%, respectively. Table 2 further describes the patient demographics. The observed MACE event prevalence of 6.6% (*n* = 91) over the follow-up period is consistent with comparable CAC-screened cohorts and provides a sufficient number of events for assessment of incremental predictive performance, where prior studies have demonstrated clinically meaningful improvements in discrimination in the range of *Δ*C-index of 0.01–0.03 (19).

**Table 2:** Patient demographics.

Characteristic	had MACE, *N* = 91^a^	no MACE, *N* = 1,289^a^	*p*-value^b^
Race			0.8
Caucasian/White	87 (96%)	1,235 (96%)	
Other/Unknown	4 (4.4%)	54 (4.2%)	
Age at screen	61 (53- 68)	58 (51- 65)	0.007
Patient sex			<0.001
Female	41 (45%)	845 (66%)	
Male	50 (55%)	444 (34%)	
Smoking status			0.2
Never	51 (56)	852 (66)	852 (66)
Quit	36 (40)	369 (29)	369 (29)
Yes	4 (4.4)	68 (5.3)	68 (5.3)
Total cholesterol	146 (129, 192)	184 (160, 211)	<0.001
HDL cholesterol	45 (38, 55)	52 (43, 63)	<0.001
Systolic blood pressure	126 (118, 131)	124 (117, 132)	0.9
Treated for blood pressure	51 (56%)	460 (36%)	<0.001
Treated for cholesterol	36 (40%)	350 (27%)	0.011
Diabetes	13 (14%)	101 (7.8%)	0.031
Hyperlipidemia	41 (45%)	388 (30%)	0.003
Heart attack	3 (3.3%)	0 (0%)	<0.001
Stroke	4 (4.4%)	0 (0%)	<0.001
Heart failure	6 (6.6%)	49 (3.8%)	0.2
Atrial fibrillation	1 (1.1%)	31 (2.4%)	0.7
Revascularization	86 (95%)	0 (0%)	<0.001

a *n* (%); Median (IQR).

b Fisher's exact test; Pearson's Chi-squared test; Wilcoxon rank sum test.

### MACE prevalence stratified by risk categories

MACE prevalence varied substantially across CAC categories:

- CAC=0: 2.9% (*n* = 22/758)
- CAC 1–99: 5.3% (*n* = 18/337)
- CAC 100–399: 12.7% (*n* = 20/157)
- CAC ≥400: 24.2% (*n* = 31/128)

Within the CAC=0 group (*n* = 758), PRS provided additional risk stratification, with MACE prevalence by low (<15th percentile), intermediate (15–85th percentile), and high (> 85th percentile) PRS:

- Low PRS (<15th percentile): 2.4% (*n* = 3/124)
- Intermediate PRS (15–85th percentile): 2.2% (*n* = 12/537)
- High PRS (>85th percentile): 7.2% (*n* = 7/97)
- *p* for trend < 0.05

When combining CAC and PRS into integrated risk categories, MACE prevalence ranged from 2.4% in the lowest-risk group (CAC=0 and PRS T1) to 20% in the highest-risk group (CAC ≥400 or PRS T3), representing an 8.33-fold gradient in absolute risk. Among individuals with CAC=0 but high PRS (>85th percentile), MACE prevalence (7.2%) was comparable to those with CAC 1–99 and low-to-intermediate PRS (6.5%), demonstrating the clinical utility of PRS for refining risk in the zero-calcium population.

Comparing the no MACE and MACE groups revealed a higher prevalence of males in the MACE group (55% vs. 34%, *p* < 0.001), with the opposite trend observed among females (45% vs. 66%). When considering age at the time of their heart screen (CACS collection), patients with MACE were older than those without MACE (61 vs. 58, *p* < 0.05). Race did not differ between these cohorts (Table 2).

Regarding smoking status, there was a trend of association towards a MACE event, albeit insignificant (*p* = 0.091). Specifically, those with a MACE event had a lower prevalence of never smokers (56% vs. 66%), a higher prevalence of former smokers (40% vs. 29%), and a lower prevalence of current smokers (4.4% vs. 5.3%). Further analysis indicated a lower median total cholesterol level in the MACE group (146 vs. 1,841 mg/dL) and lower HDL cholesterol levels (45 vs. 52 mg/dL), suggesting a correlation between lipid profiles and cardiovascular risk. Those with a MACE tended to have higher rates of diabetes (14% vs. 7.8%, *p* < 0.05) and hyperlipidemia (45% vs. 30%, *p* < 0.01). Those with a MACE event also had higher rates of statin use (40% vs. 27%) (*p* < 0.001).

Prevalence rates for systolic hypertension and diabetes were higher in the MACE group (hypertension: 76% vs. 63%, *p* = 0.04; diabetes: 27% vs. 18%, *p* = 0.03). Additionally, patients in the MACE group exhibited higher systolic and diastolic blood pressure levels, indicating the need for careful management of these conditions to reduce the risk of adverse events.

Prior revascularization was also more common in the MACE group (*p* < 0.05). A history of stroke and heart failure was more prevalent among these patients, underscoring the critical need for close monitoring of individuals with these conditions.

In the Sanford Health discovery cohort, the C-index for MACE prediction was:

- CAC score alone: 0.737 (95% CI: 0.680–0.794)
- CAC+clinical risk factors: 0.716 (95% CI: 0.654–0.778)
- CAC+PRS: 0.733 (95% CI: 0.671–0.800)
- CAC+PRS+clinical risk factors: 0.748 (95% CI: 0.690–0.807)

**Figure 1:**
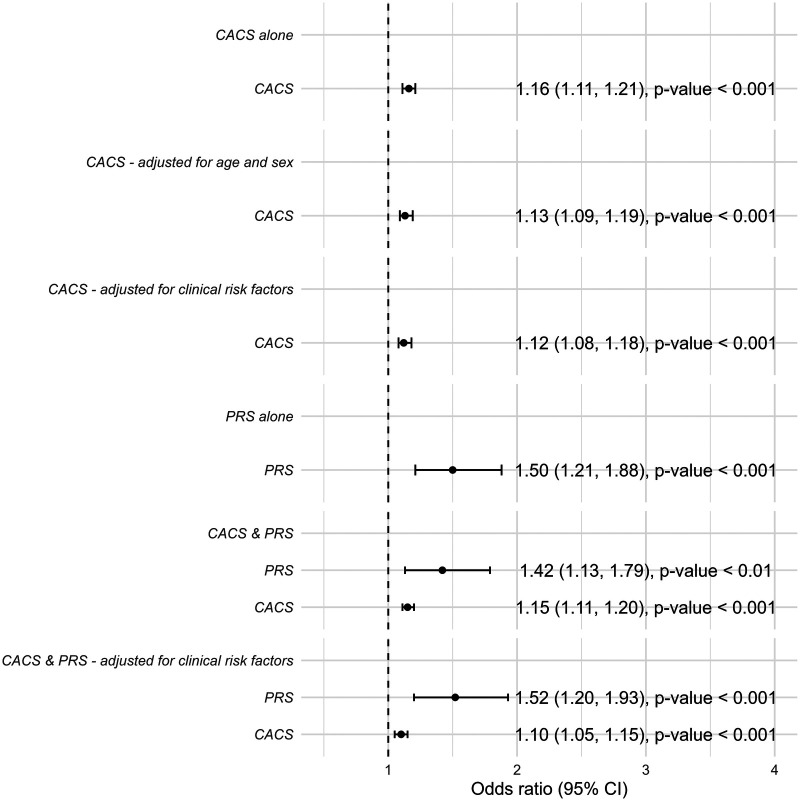
Forest plot of hazard ratios for MACE prediction across models. Forest plot showing hazard ratios (HR) and 95% confidence intervals for major adverse cardiovascular events (MACE) prediction across different risk models in the Sanford Health discovery cohort. Models are presented in order of increasing complexity: 1) CAC score alone (per 100 Agatston units); 2) CAC+age+sex; 3) CAC+clinical risk factors (age, sex, hypertension, diabetes, smoking, LDL-C); 4) PRS alone (per 1-SD increase); 5) CAC+PRS; 6) CAC+PRS+clinical risk factors; Hazard ratios are displayed with 95% confidence intervals (horizontal lines) and point estimates (squares sized proportional to precision). The vertical dashed line indicates HR = 1.0 (no effect). C-indices for each model are displayed on the right. The combined CAC+PRS+clinical risk factors model demonstrates the highest discriminatory performance (C-index=0.779).

The addition of PRS to the CAC-only model resulted in a C-index change of −0.003 (95% CI: −0.054, 0.061, *p* = 0.896). The addition of PRS to the CAC+clinical risk factors model resulted in a C-index improvement of 0.032 (95% CI: 0.716–0.748, *p* = 0.06) (Figure 1).

### Analysis of CACS and PRS in relation to MACE

Next, we sought to evaluate the distribution of patients with and without MACE based on the CACS and the PRS risk groups (<15th percentile, 15 – 85th, >85th). The results, summarized in Table 3, reveal noteworthy associations between these scores and the occurrence of MACE. In reference to CACS: Patients with low CACS (<100) accounted for 45% of the patients who experienced MACE and 82% of the patients who had no MACE. This striking difference indicates a significantly higher prevalence of MACE in the moderate (100–300) and high (>300) CACS categories (*p* < 0.001). For patients categorized with moderate CACS (100–300), 15% were in the MACE group, whereas 8.5% were in the no MACE group. Finally, the high CACS (>300) category showed an even more pronounced risk, with 40% of the MACE group represented compared to just 9.7% of the no MACE group.

**Table 3:** MACE distribution among CACS and PRS tertiles .

Characteristic	had MACE, *N* = 91^a^	no MACE, *N* = 1,289^a^	*p*-value^b^
CACS			<0.001
Low (<100)	41 (45%)	1,055 (82%)	
Moderate (100–300)	14 (15%)	109 (8.5%)	
High (>300)	36 (40%)	125 (9.7%)	
PRS (percentile)			0.006
Low (<15th)	10 (11%)	197 (15%)	
Moderate (15–85th)	57 (63%)	909 (71%)	
High (>85th)	24 (26%)	183 (14%)	

a *n* (%).

b Pearson's Chi-squared test.

In terms of PRS: when examining the PRS, we observed the following: in the low PRS category (<15th percentile), only 11% of patients were in the MACE group, contrasted with 15% in the no MACE group (*p* = 0.006), indicating a lower likelihood of adverse events. Notably, the moderate PRS (15th-85th percentile) included 63% of the MACE group vs. 71% of the no MACE group, while 26% of the MACE group fell into the high PRS category (>85th percentile), compared to 14% in the no MACE group.

Post-hoc testing revealed that patients with a “high” CAD PRS as compared to “moderate/low” were 2.2 times more likely to have a future MACE event [OR=2.16 (1.26, 3.60), *p* < 0.05]. Moreover, our analysis demonstrated a significant association between categorical PRS and the likelihood of future MACE events, with a chi-squared statistic of *χ*^2^ (2, *N* = 1,380) = 10.20, *p* < 0.05. Furthermore, a categorical CACS was found to be associated with a future MACE event, X^2^ (2, *N* = 1,380) = 84.04, *p* < 0.05.

### Regression analysis of risk factors associated with major adverse cardiovascular events: insights from CACS and PRS

Next, we aimed to evaluate the combined utility of the CACS and PRS in predicting major MACE, given the need for improved tools to stratify cardiovascular risk. The conducted multiple logistic regression analysis revealed significant associations between MACE and both CACS and PRS, as shown in Table 4. High PRS [OR = 2.15 (95% CI: 1.49–3.13), *p* < 0.05] and high CACS [OR = 4.47 (95% CI: 2.60–7.68), *p* < 0.05] were independently associated with an increased risk of future MACE. The model with only CACS and covariates explained 6.0% of the variance in MACE risk, while adding PRS increased the explained variance to 7.1%.

**Table 4:** Regression results of MACE on CACS and CAD PRS as hierarchical model.

Characteristic	CACS		PRS		CACS & PRS	
	OR (95% CI)	*p*-value	OR (95% CI)	*p*-value	OR (95% CI)	*p*-value
Sex						
Female	—		—		—	
Male	1.51 (0.95 to 2.41)	0.083	2.38 (1.52 to 3.73)	<0.001	1.68 (1.04 to 2.72)	0.032
Age	1.00 (0.97 to 1.03)	0.97	1.02 (1.00 to 1.05)	0.060	1.00 (0.98 to 1.03)	0.89
Diabetes	1.27 (0.63 to 2.39)	0.49	1.27 (0.63 to 2.39)	0.48	1.27 (0.62 to 2.42)	0.48
Hyperlipidemia	1.37 (0.86 to 2.19)	0.18	1.35 (0.84 to 2.13)	0.21	1.33 (0.82 to 2.12)	0.24
On BP treatment	1.76 (1.12 to 2.79)	0.015	2.00 (1.27 to 3.15)	0.003	1.82 (1.15 to 2.89)	0.011
Statin						
N	—		—		—	
Y	0.98 (0.59 to 1.61)	0.94	1.11 (0.67 to 1.79)	0.69	0.97 (0.58 to 1.60)	0.91
CACS	4.47 (2.60 to 7.68)	<0.001			3.98 (2.28 to 6.92)	<0.001
PRS			2.15 (1.49 to 3.13)	<0.001	1.92 (1.32 to 2.81)	<0.001

CI, confidence interval; OR, odds ratio.

When PRS and CACS were modeled jointly above and beyond covariates, males had a higher, risk of MACE compared to females [OR = 1.68 (95% CI: 1.04–2.72), *p* < 0.05], while diabetes showed a similar nonsignificant trend [OR = 1.27 (95% CI: 0.62–2.29), *p* = 0.158]. Hyperlipidemia trended toward an association with MACE [OR = 1.33 (95% CI: 0.82–2.12), *p* = 0.24]. Individually, both CACS [OR = 3.98 (95% CI: 2.28–6.92), *p* < 0.001] and PRS [OR = 1.92 (95% CI: 1.32–2.81), *p* < 0.001] were significant predictors of MACE.

### ROC curve analysis shows enhanced predictive accuracy for MACE with combined CACS and PRS

**Figure 2:**
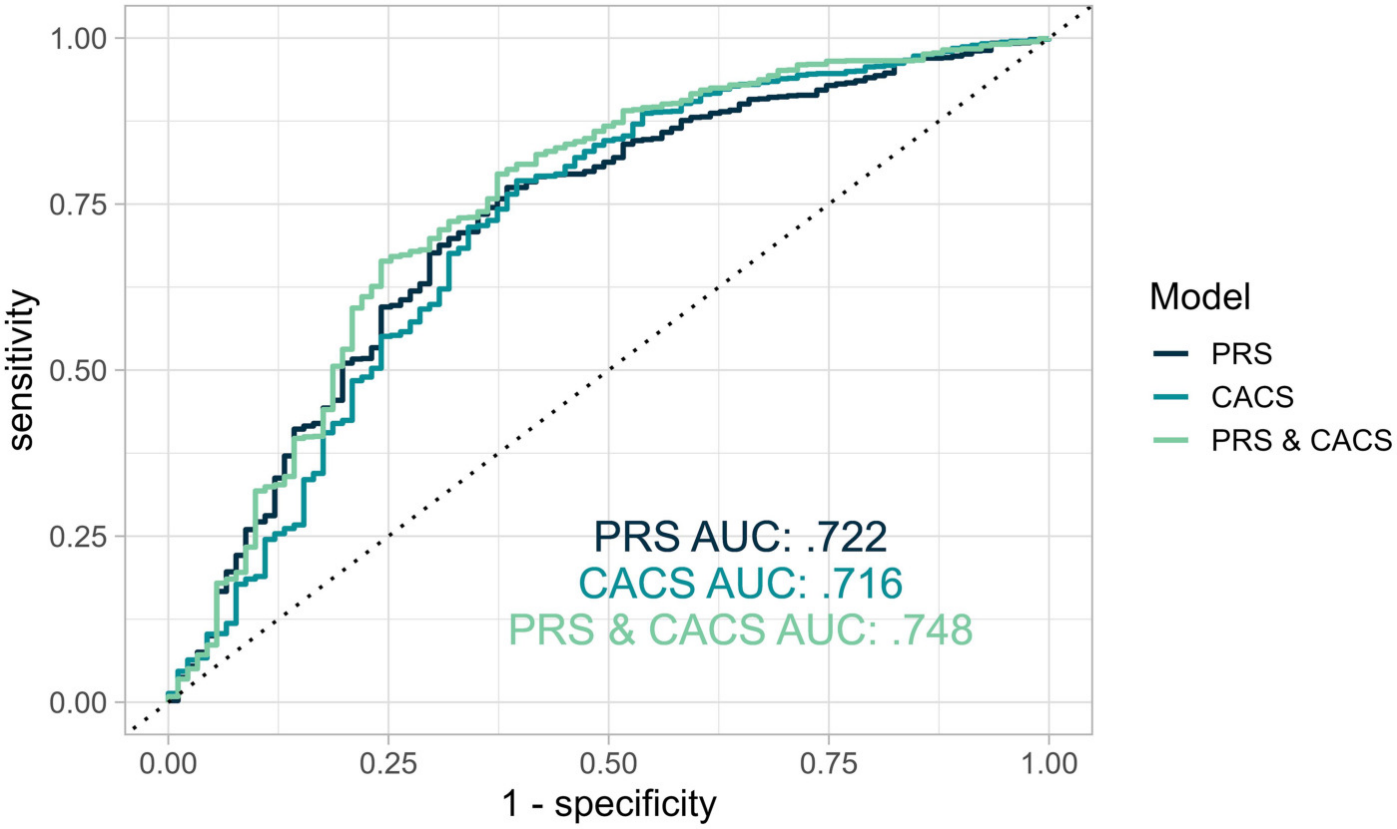
Sensitivity and specificity for MACE prediction using different testing modalities. Receiver operating characteristic (ROC) curves comparing the predictive performance of PRS alone, CACS alone, and the combined PRS+CACS model for major adverse cardiovascular events (MACE). The diagonal dashed line represents the line of no discrimination (AUC=0.50). The PRS model is represented by black curve, the CACS model by blue curve, and the PRS+CACS model by green curve respectively.

To predict MACE, we performed ROC and AUPRC curve analyses for the CACS and the PRS via logistic regression estimations above and beyond clinical risk factors. The area under the curve (AUC) and area under the precision recall curve (AUPRC) for the PRS were 0.722 and 0.9651 reflecting moderate predictive ability for MACE. Similarly, the AUC and AUPRC for the CACS were 0.7167 and 0.964 indicating comparable predictive power (Figure 2). Notably, when combining both PRS and CACS in a logistic regression model, the AUC and AUPRC increased to 0.748 and 0.968, demonstrating enhanced predictive capability, which indicates superior performance over either score alone. The combined model (PRS & CACS) having the highest AUC suggests that using both scoring systems together enhances the accuracy of predicting MACE. Furthermore, a comparison in brier scores indicated that PRS & CACS had a better fit (0.057) to actual MACE events as compared to PRS (0.059) and CACS (0.058) alone.

**Figure 3:**
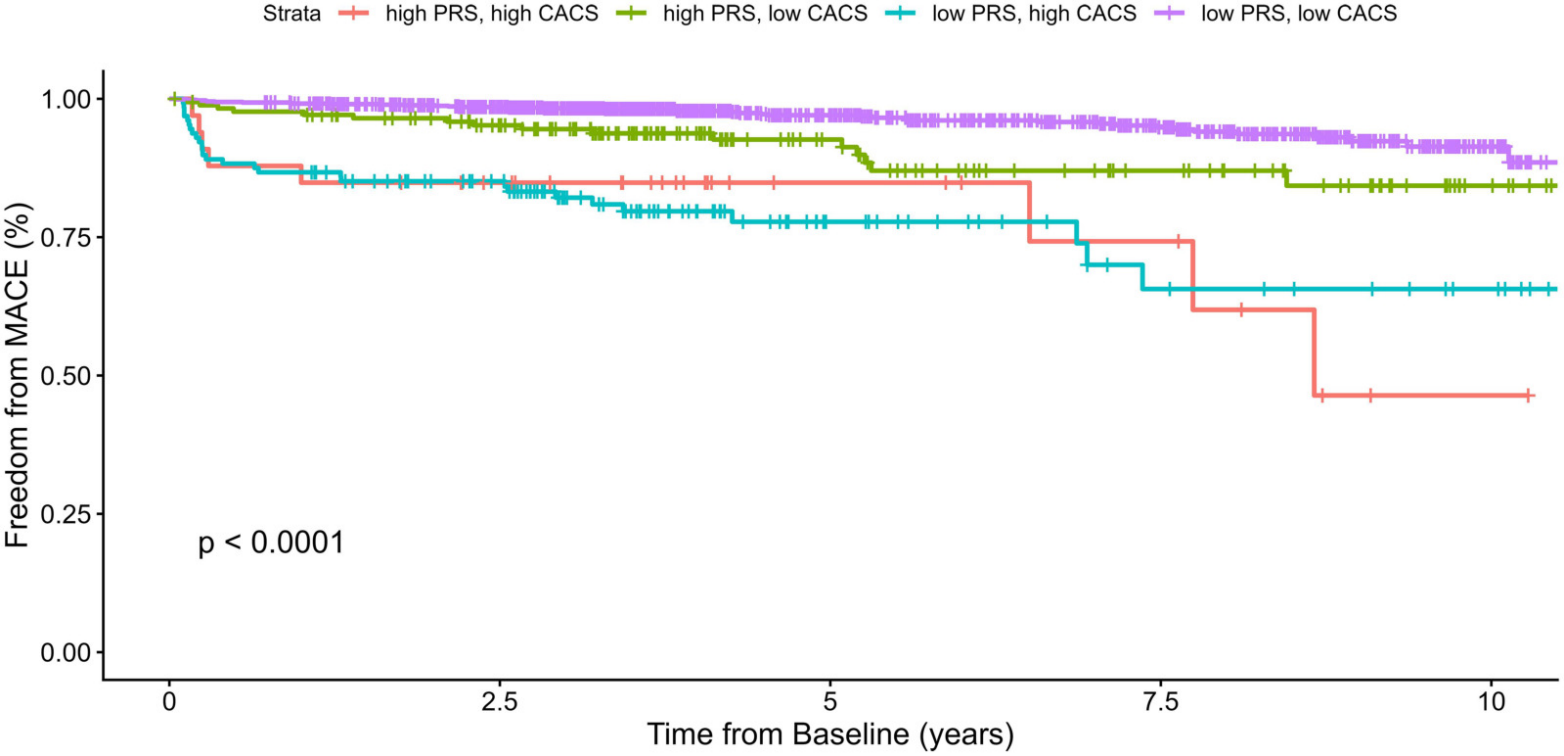
Kaplan–Meier Curves showing event-free survival for different combinations of CACS and PRS risk categories. Kaplan–Meier survival curves depicting time-to-MACE events stratified by combined risk categories of CACS and PRS. Patients were classified into four groups based on high (>85th percentile) or low (≤85th percentile) CACS and PRS values: (1) Low PRS+Low CACS (green line), (2) Low PRS+High CACS (blue line), (3) High PRS+Low CACS (orange line), and (4) High PRS+High CACS (red line). The *x*-axis represents follow-up time in years, and the *y*-axis represents the cumulative probability of remaining event-free (survival probability). Log-rank test was used to compare survival distributions across groups.

In summary, the ROC analysis illustrates that both CACS and PRS have moderate predictive capabilities for MACE, but the combination of both provides significant improvement. This supports the notion that integrating multiple risk assessment tools may enhance cardiovascular risk stratification in clinical practice (Figure 3).

### Combined risk score categories & time-to-MACE

Time-to-MACE event was regressed via each combination of four stratified risk categories (high PRS, high CACS; high PRS, low CACS; low PRS, high CACS; low PRS, low CACS). Results revealed that via a statistically significant effect of this categorical representation and hazard risk of MACE (*p* < 0.001) (Figure 3). Kaplan-Meyer curves demonstrate that patients with a high PRS and a high CACS had the lowest survival rate amongst all levels of high and low risk amongst the 2 risk scores.

## Discussion

### Application of CACS

The relationship between Coronary Artery Calcium Score (CACS) and Major Adverse Cardiovascular Events (MACE) has been extensively studied and well established in literature. In the landmark MESA study, Budoff et al. demonstrated a strong, graded association between CACS and the 10-year risk of incident Atherosclerotic Cardiovascular Disease (ASCVD), similar to its correlation with coronary heart disease (CHD) (6). Importantly, this association remains consistent across various demographic groups, including race, age, and sex, highlighting CACS as a reliable predictor of ASCVD across diverse patient populations (6). Our results align with these findings, showing a statistically significant association between CACS tertiles and the occurrence of MACE. Specifically, individuals with high CACS were 6.1 times more likely to experience future MACE compared to those with moderate or low CACS.

For individuals at the low extreme of traditional risk factor burden, Wang et al. found that incorporating CACS into pretest probability models (8), such as GEM and CONFIRM, led to more accurate risk estimation. This was evident through improvements in integrated discrimination and net reclassification. Receiver operating characteristic (ROC) curves further highlighted that integrating CACS into GEM significantly enhanced risk assessment compared to using the models independently. This increased accuracy can help avoid unnecessary additional testing in low-risk patients, streamlining clinical decision-making (8).

CAC is not benign even in younger populations. In a study, following individuals aged between 32 and 46 years, the presence of CACS was associated with an increased risk of both fatal and nonfatal CHD over 12.5 years of follow-up. Adults aged 50 years or younger, even with very low CACS scores, demonstrated an increased risk of clinical CHD, cardiovascular disease (CVD), and mortality, reinforcing the importance of CACS in younger individuals (16).

In line with these findings, clinical practice guidelines from the ACC/AHA support the selective use of CACS scoring to guide treatment decisions in primary prevention. This strategy aims to improve risk assessment and better identify individuals at both high and very low risk for ASCVD (20–23) Furthermore, CACS is utilized in the reclassification of patients with intermediate 10-year ASCVD risk (7.5% to <20%), categorizing them into two groups: “no statin” (CACS score=0) or “initiate statin” (CACS score ≥100 or ≥75th percentile by age and sex) (24). This recommendation is supported by the 2018 multi-society guidelines on cholesterol management and validated by retrospective analyses of the Framingham Heart Study and MESA, where a significant proportion of patients were downgraded from intermediate risk to the “no statin” group. These analyses also confirm that patients with CACS >100 or at the 75th percentile have a higher long-term risk of ASCVD (24–27).

### Clinical utility of CAD PRS

Polygenic risk scores (PRSs) have demonstrated strong predictive capabilities for subclinical coronary atherosclerosis and offer an early indication of cardiovascular events, independent of established clinical and demographic risk factors (11). By analyzing extensive genetic data, genomic risk scores have been developed as an additional screening tool for primary prevention, complementing traditional risk prediction models. In a study by Inouye et al., the metaGRS effectively categorized individuals into distinct life-course trajectories of coronary artery disease (CAD) risk (12). Those in the top 20% of the metaGRS distribution had a 4.17 times higher likelihood of developing CAD compared to individuals in the bottom 20%. Furthermore, the genomic risk score outperformed six conventional risk factors—smoking, diabetes, hypertension, body mass index, self-reported high cholesterol, and family history—in predicting incident CAD (C-index=0.623; 95% CI: 0.615 to 0.631). Our findings also demonstrated a significant association between CAD PRS tertiles and incident MACE events, with individuals in the high PRS group being 2.2 times more likely to experience future MACE than those with moderate or low PRS.

A key advantage of genomic screening with PRS is its ability to identify high-risk individuals early in life, before the onset of comorbid risk factors or disease (13). Using large-scale biobank data from the FINRISK study, Mars et al. demonstrated that the application of PRS for disease prediction significantly improved net reclassification, particularly for early-onset coronary heart disease (CHD). Furthermore, individuals with a high PRS had a 21% to 38% higher lifetime risk and experienced disease onset 4 to 9 years earlier than those with an average PRS. This effect was not limited to CHD, but extended to four other common diseases as well (28).

PRSs are not only effective as a standalone tool for predicting atherosclerotic cardiovascular disease (ASCVD) but also show promise when integrated with established prediction tools such as the AHA/ACC pooled cohort equations (PCE) or UK QRISK3. In a study of 60,000 individuals from the UK Biobank, integrated risk tools (IRTs) that combined PRS with traditional models outperformed individual tools, demonstrating superior predictive power for incident CAD and greater net reclassification (*N*RI 5.9; 95% CI, 4.7–7.0), which enables earlier intervention (15). In a larger observational study involving 352,660 individuals from the UK Biobank, more modest improvements in discriminant accuracy for incident CAD (C-index=0.02; 95% CI, 0.01 to 0.03) and reclassification benefit (NRI 4%; 95% CI, 0.031–0.049) were observed when comparing PCEs with an IRT that includes PRS (14). These findings suggest that while PRS integration with other tools shows promise, further investigation is needed before widespread clinical implementation can be confidently recommended (14, 15, 29). Nonetheless, these results invite exploration into how PRS can be optimized in combination with other screening methods for enhanced risk prediction.

### Direct comparison and interplay between PRS and CACS

Significant improvements in the prediction of incident cardiovascular events have been noted when either PRS or CACS are added to traditional risk models such as the AHA/ACC pooled cohort equations (PCE) (11). Our primary objective was to directly compare the diagnostic utility of these two modalities, both individually and in combination, to determine whether the prediction of future MACE could be enhanced. In July 2022, a scientific statement from the AHA highlighted the lack of direct comparisons between PRS and CACS, emphasizing the need for prospective studies in this area (11).

At the time of writing, Khan and colleagues presented one of the most comprehensive comparisons between PRS and CACS, exploring the changes in discrimination when either modality is added to a coronary heart disease (CHD) prediction model based on traditional risk factors (30, 31). In two cohorts, one from the United States and one from the Netherlands, comprising individuals aged 45 to 79 years, CACS was found to be superior to PRS in discriminating CHD risk. Adding both CACS and PRS to the PCE provided the best discrimination overall (C-index=0.10; 95% CI, 0.07–0.14). Additionally, it was only the incorporation of CACS, and not PRS, that led to enhanced risk discrimination and reclassification [net reclassification index (NRI) 0.19; 95% CI, 0.06–0.28] for CHD when combined with traditional risk factors (30, 31).

In our study, the comparative analysis of CACS and CAD PRS in predicting MACE revealed that when covariates were included, the areas under the curve (AUC) for CACS (AUC=0.707) and PRS (AUC=0.703) were similar, indicating comparable sensitivity and specificity for MACE prediction. This contrasts with previous findings in the literature. However, the integration of PRS with CACS resulted in an enhanced predictive ability, yielding an AUC of 0.734, which surpassed the predictive accuracy of either modality alone.

In terms of MACE events, our results demonstrated a clear correlation with higher CACS scores. Patients who experienced MACE events had an average CACS score of 527.1 ± 856.2, compared to 98.3 ± 294.3 in those without events. Similarly, higher CAD PRS values were linked to a greater frequency of MACE events, with an average score of 15.5 ± 0.62 for patients experiencing events, compared to 15.2 ± 0.61 for those without. These findings highlight the valuable contribution of both CACS and CAD PRS in predicting MACE, with the combined model showing improved performance. This suggests that integrating these two tools could offer more robust risk assessment strategies.

Moreover, the utility of combining PRS with CACS may extend to patients with no detectable coronary calcium. Al Rifai et al. demonstrated that in patients with a CACS of 0, ASCVD PRS could still identify incident ASCVD events, primarily driven by genetic variants related to stroke rather than CHD, and predominantly in women and non-Caucasian individuals (32). This suggests that incorporating PRS could provide valuable predictive insights even in situations where traditional measures like CACS fail to yield information.

### Future applications & limitations

The interplay between polygenic risk scores (PRS) and coronary artery calcium score (CACS) represents a rapidly evolving area of study, with the potential to significantly improve risk prediction and disease prevention strategies. The application of integrated risk tools (IRTs) to optimize cardiovascular risk assessment is a focus of ongoing research, and emerging findings are expected to influence future clinical practice guidelines. For instance, the ongoing ESCALATE study from Australia, which investigates the role of PRS-triaged CACS in identifying subclinical coronary artery disease (CAD), aims to further refine our understanding of how best to apply these testing modalities in clinical practice (33).

 There are several limitations to consider in our study. First, our study population was relatively small compared to larger biobank studies, and it was primarily composed of Caucasian individuals from a single hospital system in the Midwest. All three cohorts (discovery, Rotterdam Study, and MESA) included individuals who underwent CAC imaging, which may represent a selected population with higher cardiovascular risk factor burden compared to the general population. As such, the generalizability of our findings to unselected more diverse patient populations with low- and middle-income settings may be limited and requires further investigation and validation.

Secondly, the relatively small number of MACE events (*n* = 91) may raise concern about statistical power. However, the total number of MACE events and overall incidence of 6.6% is congruent with 4-year MACE incidence of 6.9% reported in the literature for patients with established atherosclerotic disease or multiple risk factors managed in routine practice (17).

Due to methodological constraints, CAD PRS was only calculated for patients who self-identified as white, and our discovery cohort was predominantly of European ancestry (96%), limiting our ability to assess PRS performance in diverse populations. Current CAD PRS models, including the one used in this study, are derived primarily from European-ancestry GWAS and show attenuated performance in non-European populations due to differences in linkage disequilibrium patterns, allele frequencies, and potentially causal variant distributions.

The use of a European-ancestry-derived GWAS for PRS construction represents both a practical necessity given current data availability and a significant limitation. While the CARDIoGRAMplusC4D GWAS remains one of the largest and most well-validated CAD GWAS, its predominantly European composition limits the transferability of the resulting PRS to non-European populations. Emerging trans-ancestry GWAS and multi-ancestry PRS methods show promise for improving performance across diverse populations, and future iterations of CAC + PRS risk models should incorporate these advances as they become available and validated.

Future work must prioritize: (a) large-scale GWAS in diverse populations to identify ancestry-specific and shared causal variants, (b) trans-ancestry meta-analyses and PRS methods that leverage shared genetic architecture while accounting for population-specific effects, (c) rigorous validation of PRS across ancestries before clinical implementation, and (d) equitable recruitment and retention of diverse populations in biobank and genetic studies.

Until ancestry-diverse PRS models are available and validated, clinicians should exercise caution when applying European-ancestry-derived PRS to patients of non-European ancestry. PRS should be considered as one component of comprehensive risk assessment rather than a standalone tool, and clinical decision-making should integrate PRS with traditional risk factors, biomarkers, and imaging in the context of individual patient characteristics and preferences.

Our study used ICD-10 codes from electronic health records for MACE ascertainment rather than formal adjudication by expert clinicians reviewing source documents. The use of ICD-10–based algorithms for cardiovascular outcome ascertainment is well established in epidemiologic research and enables large-scale longitudinal studies with comprehensive outcome capture. Prior validation studies have demonstrated high positive predictive value (PPV) for key components such as acute myocardial infarction and stroke, including work by McCormick N et al. and Kiyota Y et al., supporting the reliability of ICD-based definitions in administrative datasets (34, 35). To enhance specificity and mitigate potential misclassification, we used validated ICD-10 code algorithms with reported PPV >85% for myocardial infarction, stroke, coronary revascularization and cardiovascular death, restricted ascertainment to hospital discharge diagnoses rather than outpatient encounters, excluded codes associated with rule-out diagnoses, and confirmed cardiovascular deaths through linkage with the National Death Index.

Despite these measures, ICD-10–based outcome definitions remain subject to misclassification due to coding inaccuracies, inter-institutional variability, and incomplete clinical documentation. While PPV is generally high, sensitivity may be lower, potentially resulting in under-ascertainment of events, and composite endpoints such as MACE may be particularly susceptible to heterogeneity in coding practices. In the absence of formal adjudication, both false positives and false negatives may occur; however, such misclassification is likely to be largely non-differential with respect to PRS and coronary artery calcium burden. As a result, any bias introduced would be expected to attenuate associations toward the null, potentially underestimating the true predictive value of CACS and PRS. Nonetheless, residual or differential misclassification cannot be entirely excluded and should be considered when interpreting these findings, and future studies incorporating adjudicated outcomes in a subset of participants would further strengthen confidence in outcome ascertainment.

Another limitation is that our study did not address the role of pharmacotherapy (i.e., statins) and how such interventions may modify risk profiles, potentially confounding results.

Our study benefits from validation in two independent external cohorts (Rotterdam Study and MESA) with different geographic locations, recruitment strategies, and demographic compositions, strengthening confidence in the generalizability of our findings. However, all three cohorts are from high-income countries with access to advanced cardiovascular care, and validation in low- and middle-income settings is needed. Additionally, all cohorts included individuals who underwent CAC imaging, which may represent a selected population with higher cardiovascular risk factor burden compared to the general population. The generalizability of our findings to unselected community populations requires further investigation.

## Conclusion

This study provides valuable insights into the relationship between coronary artery disease (CAD) risk prediction models, specifically focusing on coronary artery calcium score (CACS) and polygenic risk score (PRS). By evaluating the diagnostic utility of each modality both individually and in combination, we have gained a better understanding of their effectiveness in predicting major adverse cardiovascular events (MACE). Both CACS and CAD PRS were significantly associated with future MACE events, highlighting their importance in cardiovascular risk assessment. When directly compared, CACS demonstrated superior predictive performance, especially when integrated with CAD PRS, which resulted in enhanced risk prediction capabilities. These findings suggest that combining CACS and CAD PRS could offer a more refined approach to risk assessment and improve primary prevention strategies. Nevertheless, further research and prospective studies are needed to validate these findings and explore their broader clinical implications. The continued investigation into the synergy between PRS and CACS holds great promise for advancing cardiovascular risk prediction and disease prevention.

## Data Availability

The data that supports the findings of this study are available upon reasonable request. Requests should be directed to the management team of Sanford Health, which has protocols for approving data requests, and can be submitted via the corresponding author, Dr. Tomasz Stys (tomasz.stys@sanfordhealth.org). Because of restrictions based on privacy regulations and informed consent of the participants, data cannot be made freely available in a public repository.
