# Supplementary material for: Addition of CAD polygenic risk score to coronary artery calcium score enhances prediction of MACE
Source: Front Cardiovasc Med. 2026 Jun 4;13:1814510. doi: 10.3389/fcvm.2026.1814510 (PMC13276707; doi:10.3389/fcvm.2026.1814510)
Supplement: Supplementary file 1 [file Datasheet1.docx]

| **Supplementary Table 1** LD pruned SNPs - imputed data |  |
| --- | --- |

| chr_pos | chr | pos | rsid | Risk allele | Other allele | RAF | OR | effect_size | Status of the SNP being genotyped in Sanford genotped data | Proxy for palindromic SNP with RAF 0.35-0.65 (restrict)  palindromic SNPs replaced by non-palindromic proxies | SNP to include in final GRS | GRS SNP position | GRS SNP risk allele | GRS SNP other allele |
| --- | --- | --- | --- | --- | --- | --- | --- | --- | --- | --- | --- | --- | --- | --- |
| 1:2252205 | 1 | 2252205 | rs36096196 | T | C | 0.15 | 1.05 | 0.048790164 | not genotyped | #NV | rs36096196 | 2252205 | T | C |
| 1:3325912 | 1 | 3325912 | rs2493298 | A | C | 0.14 | 1.06 | 0.058268908 | not genotyped | #NV | rs2493298 | 3325912 | A | C |
| 1:38461319 | 1 | 38461319 | rs61776719 | A | C | 0.53 | 1.04 | 0.039220713 | not genotyped | #NV | rs61776719 | 38461319 | A | C |
| 1:55496039 | 1 | 55496039 | rs11206510 | T | C | 0.85 | 1.08 | 0.076961041 | genotyped | #NV | rs11206510 | 55496039 | T | C |
| 1:55505647 | 1 | 55505647 | rs11591147 | G | T | 0.984 | 1.25 | 0.223143551 | genotyped | #NV | rs11591147 | 55505647 | G | T |
| 1:56965664 | 1 | 56965664 | rs9970807 | C | T | 0.92 | 1.13 | 0.122217633 | genotyped | #NV | rs9970807 | 56965664 | C | T |
| 1:109817192 | 1 | 109817192 | rs7528419 | A | G | 0.79 | 1.12 | 0.113328685 | genotyped | #NV | rs7528419 | 109817192 | A | G |
| 1:115753482 | 1 | 115753482 | rs11806316 | G | A | 0.63 | 1.041666667 | 0.040821995 | not genotyped | #NV | rs11806316 | 115753482 | G | A |
| 1:151762308 | 1 | 151762308 | rs11810571 | G | C | 0.849 | 1.06 | 0.058268908 | not genotyped | #NV | rs11810571 | 151762308 | G | C |
| 1:154395946 | 1 | 154395946 | rs6689306 | A | G | 0.45 | 1.06 | 0.058268908 | genotyped | #NV | rs6689306 | 154395946 | A | G |
| 1:169094459 | 1 | 169094459 | rs1892094 | C | T | 0.5 | 1.041666667 | 0.040821995 | not genotyped | #NV | rs1892094 | 169094459 | C | T |
| 1:200646073 | 1 | 200646073 | rs6700559 | C | T | 0.53 | 1.041666667 | 0.040821995 | not genotyped | #NV | rs6700559 | 200646073 | C | T |
| 1:201872264 | 1 | 201872264 | rs2820315 | T | C | 0.3 | 1.05 | 0.048790164 | not genotyped | #NV | rs2820315 | 201872264 | T | C |
| 1:210468999 | 1 | 210468999 | rs60154123 | T | C | 0.15 | 1.04 | 0.039220713 | not genotyped | #NV | rs60154123 | 210468999 | T | C |
| 1:222762709 | 1 | 222762709 | rs17464857 | T | G | 0.856 | 1.06 | 0.058268908 | not genotyped | #NV | rs17464857 | 222762709 | T | G |
| 1:222823743 | 1 | 222823743 | rs67180937 | G | T | 0.66 | 1.08 | 0.076961041 | genotyped | #NV | rs67180937 | 222823743 | G | T |
| 1:230845794 | 1 | 230845794 | rs699 | G | A | 0.42 | 1.041666667 | 0.040821995 | genotyped | #NV | rs699 | 230845794 | G | A |
| 2:19942473 | 2 | 19942473 | rs16986953 | A | G | 0.1 | 1.09 | 0.086177696 | genotyped | #NV | rs16986953 | 19942473 | A | G |
| 2:21286057 | 2 | 21286057 | rs515135 | C | T | 0.79 | 1.07 | 0.067658648 | genotyped | #NV | rs515135 | 21286057 | C | T |
| 2:44073881 | 2 | 44073881 | rs6544713 | T | C | 0.32 | 1.05 | 0.048790164 | genotyped | #NV | rs6544713 | 44073881 | T | C |
| 2:45896437 | 2 | 45896437 | rs582384 | A | C | 0.53 | 1.03 | 0.029558802 | not genotyped | #NV | rs582384 | 45896437 | A | C |
| 2:85788175 | 2 | 85788175 | rs7568458 | A | T | 0.45 | 1.06 | 0.058268908 | genotyped | rs1561198 | rs1561198 | 85809989 | T | C |
| 2:145286559 | 2 | 145286559 | rs17678683 | G | T | 0.09 | 1.1 | 0.09531018 | genotyped | #NV | rs17678683 | 145286559 | G | T |
| 2:145801461 | 2 | 145801461 | rs2252641 | C | T | 0.467 | 1.03 | 0.029558802 | genotyped | #NV | rs2252641 | 145801461 | C | T |
| 2:164957251 | 2 | 164957251 | rs12999907 | A | G | 0.82 | 1.06 | 0.058268908 | not genotyped | #NV | rs12999907 | 164957251 | A | G |
| 2:188196469 | 2 | 188196469 | rs840616 | C | T | 0.65 | 1.041666667 | 0.040821995 | genotyped | #NV | rs840616 | 188196469 | C | T |
| 2:203745885 | 2 | 203745885 | rs6725887 | C | T | 0.11 | 1.14 | 0.131028262 | genotyped | #NV | rs6725887 | 203745885 | C | T |
| 2:216291359 | 2 | 216291359 | rs17517928 | C | T | 0.75 | 1.06 | 0.058268908 | not genotyped | #NV | rs17517928 | 216291359 | C | T |
| 2:216304384 | 2 | 216304384 | rs1250229 | T | C | 0.256 | 1.072 | 0.069526063 | genotyped | #NV | rs1250229 | 216304384 | T | C |
| 2:218683154 | 2 | 218683154 | rs2571445 | A | G | 0.39 | 1.04 | 0.039220713 | not genotyped | #NV | rs2571445 | 218683154 | A | G |
| 2:227100698 | 2 | 227100698 | rs2972146 | T | G | 0.65 | 1.06 | 0.058268908 | genotyped | #NV | rs2972146 | 227100698 | T | G |
| 2:233633460 | 2 | 233633460 | rs1801251 | A | G | 0.35 | 1.06 | 0.058268908 | not genotyped | #NV | rs1801251 | 233633460 | A | G |
| 2:238223955 | 2 | 238223955 | rs11677932 | G | A | 0.68 | 1.030927835 | 0.030459207 | not genotyped | #NV | rs11677932 | 238223955 | G | A |
| 3:14928077 | 3 | 14928077 | rs748431 | G | T | 0.36 | 1.05 | 0.048790164 | not genotyped | #NV | rs748431 | 14928077 | G | T |
| 3:46688562 | 3 | 46688562 | rs7633770 | A | G | 0.41 | 1.03 | 0.029558802 | not genotyped | #NV | rs7633770 | 46688562 | A | G |
| 3:48193515 | 3 | 48193515 | rs7617773 | T | C | 0.67 | 1.04 | 0.039220713 | not genotyped | #NV | rs7617773 | 48193515 | T | C |
| 3:49448566 | 3 | 49448566 | rs7623687 | A | C | 0.855 | 1.074 | 0.071389996 | not genotyped | #NV | rs7623687 | 49448566 | A | C |
| 3:124475201 | 3 | 124475201 | rs142695226 | G | T | 0.141 | 1.071 | 0.068592791 | not genotyped | #NV | rs142695226 | 124475201 | G | T |
| 3:132257961 | 3 | 132257961 | rs10512861 | G | T | 0.86 | 1.041666667 | 0.040821995 | not genotyped | #NV | rs10512861 | 132257961 | G | T |
| 3:136069472 | 3 | 136069472 | rs667920 | T | G | 0.775 | 1.05 | 0.048790164 | not genotyped | #NV | rs667920 | 136069472 | T | G |
| 3:138122122 | 3 | 138122122 | rs9818870 | T | C | 0.14 | 1.07 | 0.067658648 | genotyped | #NV | rs9818870 | 138122122 | T | C |
| 3:153839866 | 3 | 153839866 | rs12493885 | C | G | 0.886 | 1.074 | 0.071389996 | genotyped | #NV | rs12493885 | 153839866 | C | G |
| 3:156852592 | 3 | 156852592 | rs4266144 | G | C | 0.32 | 1.030927835 | 0.030459207 | not genotyped | #NV | rs4266144 | 156852592 | G | C |
| 3:172115902 | 3 | 172115902 | rs12897 | G | A | 0.41 | 1.041666667 | 0.040821995 | not genotyped | #NV | rs12897 | 172115902 | G | A |
| 4:3449652 | 4 | 3449652 | rs16844401 | A | G | 0.07 | 1.07 | 0.067658648 | genotyped | #NV | rs16844401 | 3449652 | A | G |
| 4:57838583 | 4 | 57838583 | rs17087335 | T | G | 0.21 | 1.06 | 0.058268908 | genotyped | #NV | rs17087335 | 57838583 | T | G |
| 4:77416627 | 4 | 77416627 | rs12500824 | A | G | 0.36 | 1.04 | 0.039220713 | not genotyped | #NV | rs12500824 | 77416627 | A | G |
| 4:81181072 | 4 | 81181072 | rs10857147 | T | A | 0.275 | 1.056 | 0.054488185 | not genotyped | #NV | rs10857147 | 81181072 | T | A |
| 4:82587050 | 4 | 82587050 | rs11099493 | A | G | 0.69 | 1.04 | 0.039220713 | genotyped | #NV | rs11099493 | 82587050 | A | G |
| 4:96117371 | 4 | 96117371 | rs3775058 | A | T | 0.234 | 1.04 | 0.039220713 | not genotyped | #NV | rs3775058 | 96117371 | A | T |
| 4:120909501 | 4 | 120909501 | rs7678555 | C | A | 0.301 | 1.049 | 0.047837329 | not genotyped | #NV | rs7678555 | 120909501 | C | A |
| 4:148281001 | 4 | 148281001 | rs4593108 | C | G | 0.8 | 1.07 | 0.067658648 | genotyped | #NV | rs4593108 | 148281001 | C | G |
| 4:156639888 | 4 | 156639888 | rs72689147 | G | T | 0.82 | 1.07 | 0.067658648 | genotyped | #NV | rs72689147 | 156639888 | G | T |
| 4:169687725 | 4 | 169687725 | rs7696431 | T | G | 0.51 | 1.035619709 | 0.035 | not genotyped | #NV | rs7696431 | 169687725 | T | G |
| 5:9556694 | 5 | 9556694 | rs1508798 | T | C | 0.81 | 1.05 | 0.048790164 | not genotyped | #NV | rs1508798 | 9556694 | T | C |
| 5:55860781 | 5 | 55860781 | rs3936511 | G | A | 0.18 | 1.041666667 | 0.040821995 | not genotyped | #NV | rs3936511 | 55860781 | G | A |
| 5:121413208 | 5 | 121413208 | rs1800449 | T | C | 0.17 | 1.07 | 0.067658648 | genotyped | #NV | rs1800449 | 121413208 | T | C |
| 5:131667353 | 5 | 131667353 | rs273909 | G | A | 0.12 | 1.06 | 0.058268908 | not genotyped | #NV | rs273909 | 131667353 | G | A |
| 5:142516897 | 5 | 142516897 | rs246600 | T | C | 0.48 | 1.05 | 0.048790164 | genotyped | #NV | rs246600 | 142516897 | T | C |
| 6:1617143 | 6 | 1617143 | rs9501744 | C | T | 0.87 | 1.052631579 | 0.051293294 | not genotyped | #NV | rs9501744 | 1617143 | C | T |
| 6:2933355 | 6 | 2933355 | rs318450 | A | G | 0.44 | 1.07 | 0.067658648 | not genotyped | #NV | rs318450 | 2933355 | A | G |
| 6:11327021 | 6 | 11327021 | rs742115 | C | T | 0.484 | 1.04 | 0.039220713 | genotyped | #NV | rs742115 | 11327021 | C | T |
| 6:11774583 | 6 | 11774583 | rs6903956 | A | G | 0.35 | 1.65 | 0.500775288 | genotyped | #NV | rs6903956 | 11774583 | A | G |
| 6:12903957 | 6 | 12903957 | rs9349379 | G | A | 0.43 | 1.14 | 0.131028262 | genotyped | #NV | rs9349379 | 12903957 | G | A |
| 6:22612629 | 6 | 22612629 | rs6909752 | A | G | 0.351 | 1.051 | 0.049742092 | not genotyped | #NV | rs6909752 | 22612629 | A | G |
| 6:31888367 | 6 | 31888367 | rs3130683 | T | C | 0.86 | 1.08 | 0.076961041 | genotyped | #NV | rs3130683 | 31888367 | T | C |
| 6:34769765 | 6 | 34769765 | rs4472337 | T | C | 0.155 | 1.06 | 0.058268908 | not genotyped | #NV | rs4472337 | 34769765 | T | C |
| 6:35034800 | 6 | 35034800 | rs17609940 | G | C | 0.82 | 1.03 | 0.029558802 | genotyped | #NV | rs17609940 | 35034800 | G | C |
| 6:36638636 | 6 | 36638636 | rs1321309 | A | G | 0.49 | 1.03 | 0.029558802 | genotyped | #NV | rs1321309 | 36638636 | A | G |
| 6:39134099 | 6 | 39134099 | rs56336142 | T | C | 0.81 | 1.07 | 0.067658648 | genotyped | #NV | rs56336142 | 39134099 | T | C |
| 6:43758873 | 6 | 43758873 | rs6905288 | A | G | 0.57 | 1.04602786 | 0.045 | genotyped | #NV | rs6905288 | 43758873 | A | G |
| 6:57160572 | 6 | 57160572 | rs9367716 | G | T | 0.68 | 1.041666667 | 0.040821995 | not genotyped | #NV | rs9367716 | 57160572 | G | T |
| 6:82612271 | 6 | 82612271 | rs4613862 | A | C | 0.53 | 1.03 | 0.029558802 | not genotyped | #NV | rs4613862 | 82612271 | A | C |
| 6:126717064 | 6 | 126717064 | rs1591805 | A | G | 0.49 | 1.04 | 0.039220713 | not genotyped | #NV | rs1591805 | 126717064 | A | G |
| 6:134173151 | 6 | 134173151 | rs12202017 | A | G | 0.7 | 1.07 | 0.067658648 | not genotyped | #NV | rs12202017 | 134173151 | A | G |
| 6:150997401 | 6 | 150997401 | rs17080091 | C | T | 0.92 | 1.052631579 | 0.051293294 | not genotyped | #NV | rs17080091 | 150997401 | C | T |
| 6:160863532 | 6 | 160863532 | rs2048327 | C | T | 0.366 | 1.06 | 0.058268908 | genotyped | #NV | rs2048327 | 160863532 | C | T |
| 6:160961137 | 6 | 160961137 | rs3798220 | C | T | 0.019 | 1.49 | 0.39877612 | genotyped | #NV | rs3798220 | 160961137 | C | T |
| 6:161005610 | 6 | 161005610 | rs55730499 | T | C | 0.06 | 1.37 | 0.31481074 | not genotyped | #NV | rs55730499 | 161005610 | T | C |
| 6:161143608 | 6 | 161143608 | rs4252120 | T | C | 0.729 | 1.04 | 0.039220713 | genotyped | #NV | rs4252120 | 161143608 | T | C |
| 7:1937261 | 7 | 1937261 | rs10267593 | G | A | 0.8 | 1.041666667 | 0.040821995 | not genotyped | #NV | rs10267593 | 1937261 | G | A |
| 7:12261911 | 7 | 12261911 | rs11509880 | A | G | 0.36 | 1.037693021 | 0.037 | not genotyped | #NV | rs11509880 | 12261911 | A | G |
| 7:19049388 | 7 | 19049388 | rs2107595 | A | G | 0.2 | 1.08 | 0.076961041 | not genotyped | #NV | rs2107595 | 19049388 | A | G |
| 7:45077978 | 7 | 45077978 | rs2107732 | G | A | 0.913 | 1.063829787 | 0.061875404 | not genotyped | #NV | rs2107732 | 45077978 | G | A |
| 7:107176780 | 7 | 107176780 | rs112370447 | T | C | 0.28 | 1.05 | 0.048790164 | not genotyped | #NV | rs112370447 | 107176780 | T | C |
| 7:107244545 | 7 | 107244545 | rs10953541 | C | T | 0.78 | 1.05 | 0.048790164 | genotyped | #NV | rs10953541 | 107244545 | C | T |
| 7:129663496 | 7 | 129663496 | rs11556924 | C | T | 0.69 | 1.08 | 0.076961041 | genotyped | #NV | rs11556924 | 129663496 | C | T |
| 7:139757136 | 7 | 139757136 | rs10237377 | G | T | 0.65 | 1.052631579 | 0.051293294 | genotyped | #NV | rs10237377 | 139757136 | G | T |
| 7:150690176 | 7 | 150690176 | rs3918226 | T | C | 0.06 | 1.14 | 0.131028262 | genotyped | #NV | rs3918226 | 150690176 | T | C |
| 8:18286997 | 8 | 18286997 | rs6997340 | T | C | 0.31 | 1.036655846 | 0.036 | not genotyped | #NV | rs6997340 | 18286997 | T | C |
| 8:19813180 | 8 | 19813180 | rs264 | G | A | 0.85 | 1.06 | 0.058268908 | genotyped | #NV | rs264 | 19813180 | G | A |
| 8:22033615 | 8 | 22033615 | rs6984210 | G | C | 0.06 | 1.086956522 | 0.083381609 | not genotyped | #NV | rs6984210 | 22033615 | G | C |
| 8:106565414 | 8 | 106565414 | rs10093110 | G | A | 0.58 | 1.030927835 | 0.030459207 | not genotyped | #NV | rs10093110 | 106565414 | G | A |
| 8:126490972 | 8 | 126490972 | rs2954029 | A | T | 0.55 | 1.04 | 0.039220713 | genotyped | rs2980860 | rs2980860 | 126485337 | A | G |
| 9:22003790 | 9 | 22003790 | rs3217989 | T | C | 0.76 | 5.263157895 | 1.660731207 | not genotyped | #NV | rs3217989 | 22003790 | T | C |
| 9:22125503 | 9 | 22125503 | rs1333049 | C | G | 0.47 | 1.23 | 0.207014169 | genotyped | rs10757279 | rs10757279 | 22124630 | G | A |
| 9:110517794 | 9 | 110517794 | rs944172 | C | T | 0.28 | 1.041666667 | 0.040821995 | not genotyped | #NV | rs944172 | 110517794 | C | T |
| 9:113169775 | 9 | 113169775 | rs111245230 | C | T | 0.036 | 1.12 | 0.113328685 | genotyped | #NV | rs111245230 | 113169775 | C | T |
| 9:124420173 | 9 | 124420173 | rs885150 | C | T | 0.27 | 1.036655846 | 0.036 | not genotyped | #NV | rs885150 | 124420173 | C | T |
| 9:136141870 | 9 | 136141870 | rs2519093 | T | C | 0.19 | 1.08 | 0.076961041 | not genotyped | #NV | rs2519093 | 136141870 | T | C |
| 10:12303813 | 10 | 12303813 | rs61848342 | C | T | 0.36 | 1.041666667 | 0.040821995 | not genotyped | #NV | rs61848342 | 12303813 | C | T |
| 10:30323892 | 10 | 30323892 | rs2487928 | A | G | 0.42 | 1.06 | 0.058268908 | genotyped | #NV | rs2487928 | 30323892 | A | G |
| 10:44480811 | 10 | 44480811 | rs1870634 | G | T | 0.64 | 1.08 | 0.076961041 | genotyped | #NV | rs1870634 | 44480811 | G | T |
| 10:44753867 | 10 | 44753867 | rs501120 | T | C | 0.834 | 1.07 | 0.067658648 | genotyped | #NV | rs501120 | 44753867 | T | C |
| 10:82251514 | 10 | 82251514 | rs17680741 | T | C | 0.72 | 1.048122009 | 0.047 | not genotyped | #NV | rs17680741 | 82251514 | T | C |
| 10:91002927 | 10 | 91002927 | rs1412444 | T | C | 0.37 | 1.07 | 0.067658648 | genotyped | #NV | rs1412444 | 91002927 | T | C |
| 10:104604916 | 10 | 104604916 | rs11191416 | T | G | 0.87 | 1.08 | 0.076961041 | genotyped | #NV | rs11191416 | 104604916 | T | G |
| 10:105693644 | 10 | 105693644 | rs4918072 | A | G | 0.27 | 1.04 | 0.039220713 | not genotyped | #NV | rs4918072 | 105693644 | A | G |
| 10:124237612 | 10 | 124237612 | rs4752700 | G | A | 0.45 | 1.030927835 | 0.030459207 | not genotyped | #NV | rs4752700 | 124237612 | G | A |
| 11:9751196 | 11 | 9751196 | rs10840293 | A | G | 0.55 | 1.06 | 0.058268908 | not genotyped | #NV | rs10840293 | 9751196 | A | G |
| 11:10284499 | 11 | 10284499 | rs201267813 | GA | G | 0.071 | 1.05 | 0.048790164 | not genotyped | #NV | rs201267813 | 10284499 | GA | G |
| 11:10745394 | 11 | 10745394 | rs11042937 | G | T | 0.49 | 1.05 | 0.048790164 | genotyped | #NV | rs11042937 | 10745394 | G | T |
| 11:13303071 | 11 | 13303071 | rs3993105 | T | C | 0.704 | 1.048 | 0.046883586 | not genotyped | #NV | rs3993105 | 13303071 | T | C |
| 11:43696917 | 11 | 43696917 | rs7116641 | G | T | 0.31 | 1.030927835 | 0.030459207 | not genotyped | #NV | rs7116641 | 43696917 | G | T |
| 11:65349063 | 11 | 65349063 | rs3741380 | A | G | 0.476 | 1.04 | 0.039220713 | not genotyped | #NV | rs3741380 | 65349063 | A | G |
| 11:65391317 | 11 | 65391317 | rs12801636 | G | A | 0.77 | 1.052631579 | 0.051293294 | genotyped | #NV | rs12801636 | 65391317 | G | A |
| 11:75274150 | 11 | 75274150 | rs590121 | T | G | 0.3 | 1.05 | 0.048790164 | not genotyped | #NV | rs590121 | 75274150 | T | G |
| 11:100624599 | 11 | 100624599 | rs7947761 | G | A | 0.28 | 1.041666667 | 0.040821995 | not genotyped | #NV | rs7947761 | 100624599 | G | A |
| 11:103673277 | 11 | 103673277 | rs2128739 | A | C | 0.32 | 1.07 | 0.067658648 | genotyped | #NV | rs2128739 | 103673277 | A | C |
| 11:116648917 | 11 | 116648917 | rs964184 | G | C | 0.18 | 1.05 | 0.048790164 | genotyped | #NV | rs964184 | 116648917 | G | C |
| 12:7175872 | 12 | 7175872 | rs11838267 | T | C | 0.87 | 1.05 | 0.048790164 | not genotyped | #NV | rs11838267 | 7175872 | T | C |
| 12:20220033 | 12 | 20220033 | rs10841443 | G | C | 0.67 | 1.05 | 0.048790164 | not genotyped | #NV | rs10841443 | 20220033 | G | C |
| 12:57527283 | 12 | 57527283 | rs11172113 | C | T | 0.41 | 1.06 | 0.058268908 | genotyped | #NV | rs11172113 | 57527283 | C | T |
| 12:57843711 | 12 | 57843711 | rs2229357 | G | A | 0.764 | 1.05 | 0.048790164 | not genotyped | #NV | rs2229357 | 57843711 | G | A |
| 12:90008959 | 12 | 90008959 | rs2681472 | G | A | 0.2 | 1.08 | 0.076961041 | genotyped | #NV | rs2681472 | 90008959 | G | A |
| 12:95355541 | 12 | 95355541 | rs7306455 | G | A | 0.9 | 1.052631579 | 0.051293294 | genotyped | #NV | rs7306455 | 95355541 | G | A |
| 12:111884608 | 12 | 111884608 | rs3184504 | T | C | 0.42 | 1.07 | 0.067658648 | genotyped | #NV | rs3184504 | 111884608 | T | C |
| 12:118265441 | 12 | 118265441 | rs11830157 | G | T | 0.36 | 1.12 | 0.113328685 | not genotyped | #NV | rs11830157 | 118265441 | G | T |
| 12:121416988 | 12 | 121416988 | rs2244608 | G | A | 0.335 | 1.053 | 0.051643233 | genotyped | #NV | rs2244608 | 121416988 | G | A |
| 12:124427306 | 12 | 124427306 | rs11057401 | T | A | 0.69 | 1.06 | 0.058268908 | not genotyped | #NV | rs11057401 | 124427306 | T | A |
| 12:125307053 | 12 | 125307053 | rs11057830 | A | G | 0.16 | 1.07 | 0.067658648 | genotyped | #NV | rs11057830 | 125307053 | A | G |
| 13:28973621 | 13 | 28973621 | rs9319428 | A | G | 0.31 | 1.04 | 0.039220713 | genotyped | #NV | rs9319428 | 28973621 | A | G |
| 13:33058333 | 13 | 33058333 | rs9591012 | G | A | 0.661 | 1.041666667 | 0.040821995 | not genotyped | #NV | rs9591012 | 33058333 | G | A |
| 13:110818102 | 13 | 110818102 | rs11617955 | T | A | 0.882 | 1.086956522 | 0.083381609 | genotyped | #NV | rs11617955 | 110818102 | T | A |
| 13:110960712 | 13 | 110960712 | rs4773144 | G | A | 0.437 | 1.04 | 0.039220713 | genotyped | #NV | rs4773144 | 110960712 | G | A |
| 13:111040681 | 13 | 111040681 | rs11838776 | A | G | 0.26 | 1.07 | 0.067658648 | genotyped | #NV | rs11838776 | 111040681 | A | G |
| 13:111049623 | 13 | 111049623 | rs9515203 | T | C | 0.752 | 1.06 | 0.058268908 | genotyped | #NV | rs9515203 | 111049623 | T | C |
| 13:113631780 | 13 | 113631780 | rs1317507 | A | C | 0.26 | 1.04 | 0.039220713 | not genotyped | #NV | rs1317507 | 113631780 | A | C |
| 14:58794001 | 14 | 58794001 | rs2145598 | G | A | 0.42 | 1.030927835 | 0.030459207 | not genotyped | #NV | rs2145598 | 58794001 | G | A |
| 14:94838142 | 14 | 94838142 | rs112635299 | G | T | 0.98 | 1.149425287 | 0.139262067 | genotyped | #NV | rs112635299 | 94838142 | G | T |
| 14:100145710 | 14 | 100145710 | rs10139550 | G | C | 0.42 | 1.06 | 0.058268908 | not genotyped | rs2895811 | rs2895811 | 100133942 | C | T |
| 15:65024204 | 15 | 65024204 | rs6494488 | A | G | 0.82 | 1.052631579 | 0.051293294 | not genotyped | #NV | rs6494488 | 65024204 | A | G |
| 15:67455630 | 15 | 67455630 | rs56062135 | C | T | 0.79 | 1.07 | 0.067658648 | genotyped | #NV | rs56062135 | 67455630 | C | T |
| 15:79124475 | 15 | 79124475 | rs4468572 | C | T | 0.59 | 1.08 | 0.076961041 | not genotyped | #NV | rs4468572 | 79124475 | C | T |
| 15:89574218 | 15 | 89574218 | rs8042271 | G | A | 0.9 | 1.1 | 0.09531018 | not genotyped | #NV | rs8042271 | 89574218 | G | A |
| 15:91416550 | 15 | 91416550 | rs17514846 | A | C | 0.44 | 1.05 | 0.048790164 | genotyped | #NV | rs17514846 | 91416550 | A | C |
| 15:96146414 | 15 | 96146414 | rs17581137 | A | C | 0.75 | 1.04 | 0.039220713 | not genotyped | #NV | rs17581137 | 96146414 | A | C |
| 16:56995236 | 16 | 56995236 | rs1800775 | A | C | 0.51 | 1.06 | 0.058268908 | genotyped | #NV | rs1800775 | 56995236 | A | C |
| 16:72130815 | 16 | 72130815 | rs1050362 | A | C | 0.38 | 1.04 | 0.039220713 | not genotyped | #NV | rs1050362 | 72130815 | A | C |
| 16:75387533 | 16 | 75387533 | rs3851738 | C | G | 0.6 | 1.05 | 0.048790164 | not genotyped | rs11643209 | rs11643209 | 75331044 | G | T |
| 16:81906423 | 16 | 81906423 | rs7199941 | A | G | 0.4 | 1.04 | 0.039220713 | not genotyped | #NV | rs7199941 | 81906423 | A | G |
| 16:83045790 | 16 | 83045790 | rs7500448 | A | G | 0.752 | 1.061 | 0.05921186 | not genotyped | #NV | rs7500448 | 83045790 | A | G |
| 17:2126504 | 17 | 2126504 | rs216172 | C | G | 0.35 | 1.05 | 0.048790164 | genotyped | rs1231206 | rs1231206 | 2125605 | A | G |
| 17:17543722 | 17 | 17543722 | rs12936587 | G | A | 0.61 | 1.03 | 0.029558802 | genotyped | #NV | rs12936587 | 17543722 | G | A |
| 17:27941886 | 17 | 27941886 | rs13723 | G | A | 0.49 | 1.041666667 | 0.040821995 | not genotyped | #NV | rs13723 | 27941886 | G | A |
| 17:30033514 | 17 | 30033514 | rs76954792 | T | C | 0.22 | 1.04 | 0.039220713 | not genotyped | #NV | rs76954792 | 30033514 | T | C |
| 17:40257163 | 17 | 40257163 | rs2074158 | C | T | 0.18 | 1.052631579 | 0.051293294 | genotyped | #NV | rs2074158 | 40257163 | C | T |
| 17:45013271 | 17 | 45013271 | rs17608766 | C | T | 0.14 | 1.07 | 0.067658648 | genotyped | #NV | rs17608766 | 45013271 | C | T |
| 17:46988597 | 17 | 46988597 | rs46522 | T | C | 0.51 | 1.04 | 0.039220713 | genotyped | #NV | rs46522 | 46988597 | T | C |
| 17:47123423 | 17 | 47123423 | rs4643373 | T | C | 0.724 | 1.05 | 0.048790164 | genotyped | #NV | rs4643373 | 47123423 | T | C |
| 17:59013488 | 17 | 59013488 | rs7212798 | C | T | 0.15 | 1.08 | 0.076961041 | not genotyped | #NV | rs7212798 | 59013488 | C | T |
| 17:62387091 | 17 | 62387091 | rs1867624 | T | C | 0.61 | 1.041666667 | 0.040821995 | not genotyped | #NV | rs1867624 | 62387091 | T | C |
| 18:47229717 | 18 | 47229717 | rs9964304 | C | A | 0.28 | 1.041666667 | 0.040821995 | not genotyped | #NV | rs9964304 | 47229717 | C | A |
| 18:57838401 | 18 | 57838401 | rs663129 | A | G | 0.26 | 1.06 | 0.058268908 | not genotyped | #NV | rs663129 | 57838401 | A | G |
| 19:8429323 | 19 | 8429323 | rs116843064 | G | A | 0.98 | 1.17 | 0.157003749 | genotyped | #NV | rs116843064 | 8429323 | G | A |
| 19:11163601 | 19 | 11163601 | rs1122608 | G | T | 0.752 | 1.06 | 0.058268908 | genotyped | #NV | rs1122608 | 11163601 | G | T |
| 19:11188247 | 19 | 11188247 | rs56289821 | G | A | 0.9 | 1.14 | 0.131028262 | genotyped | #NV | rs56289821 | 11188247 | G | A |
| 19:17855763 | 19 | 17855763 | rs73015714 | G | C | 0.2 | 1.063829787 | 0.061875404 | not genotyped | #NV | rs73015714 | 17855763 | G | C |
| 19:32882020 | 19 | 32882020 | rs12976411 | A | T | 0.91 | 1.49 | 0.39877612 | genotyped | #NV | rs12976411 | 32882020 | A | T |
| 19:33386556 | 19 | 33386556 | rs10417115 | C | T | 0.061 | 1.07 | 0.067658648 | not genotyped | #NV | rs10417115 | 33386556 | C | T |
| 19:41854534 | 19 | 41854534 | rs8108632 | T | A | 0.488 | 1.049 | 0.047837329 | not genotyped | rs4803455 | rs4803455 | 41851509 | C | A |
| 19:45422946 | 19 | 45422946 | rs4420638 | G | A | 0.17 | 1.1 | 0.09531018 | not genotyped | #NV | rs4420638 | 45422946 | G | A |
| 19:46190268 | 19 | 46190268 | rs1964272 | G | A | 0.51 | 1.045 | 0.044016885 | not genotyped | #NV | rs1964272 | 46190268 | G | A |
| 20:33764554 | 20 | 33764554 | rs867186 | A | G | 0.89 | 1.075268817 | 0.072570693 | genotyped | #NV | rs867186 | 33764554 | A | G |
| 20:39924279 | 20 | 39924279 | rs6102343 | A | G | 0.25 | 1.04 | 0.039220713 | not genotyped | #NV | rs6102343 | 39924279 | A | G |
| 20:44586023 | 20 | 44586023 | rs3827066 | T | C | 0.14 | 1.04 | 0.039220713 | not genotyped | #NV | rs3827066 | 44586023 | T | C |
| 20:57714025 | 20 | 57714025 | rs260020 | T | C | 0.13 | 1.05 | 0.048790164 | not genotyped | #NV | rs260020 | 57714025 | T | C |
| 21:30533076 | 21 | 30533076 | rs2832227 | G | A | 0.18 | 1.041666667 | 0.040821995 | not genotyped | #NV | rs2832227 | 30533076 | G | A |
| 21:35593827 | 21 | 35593827 | rs28451064 | A | G | 0.12 | 1.14 | 0.131028262 | genotyped | #NV | rs28451064 | 35593827 | A | G |
| 22:24658858 | 22 | 24658858 | rs180803 | G | T | 0.97 | 1.2 | 0.182321557 | not genotyped | #NV | rs180803 | 24658858 | G | T |
| 11:5701074 | 11 | 5701074 | rs11601507 | A | C | 0.07 | 1.038135689 | 0.037426498 | no sufficient proxy | #NV | rs11601507 | 5701074 | A | C |

**Supplementary Tabele 2** Proxy lookup - GSA chip.

| CAD SNPs for GRS calculation using genotyped data, based on GSA chip  **Column C3: SNPs used in final GRS calculation. Replace the published SNPs that are not genotyped by non-palindromic proxies**  Column 4-6: additional proxies | | | | | | |
| --- | --- | --- | --- | --- | --- | --- |
| SNPs from literature | genotyped | selected | proxy1 | proxy2 | proxy3 |  |
| 1:2252205 | NO | 1:2251160 | 1:2225284 |  |  |  |
| 1:3325912 | NO | 1:3328659 | 1:3313282 | 1:3308498 |  |  |
| 1:38461319 | NO | 1:38456106 | 1:38453576 | 1:38449910 | 1:38401933 |  |
| 1:55496039 | YES | 1:55496039 | 1:55487648 |  |  |  |
| 1:55505647 | YES | 1:55505647 |  |  |  |  |
| 1:56965664 | YES | 1:56965664 | 1:56966350 | 1:56962821 | 1:56986978 |  |
| 1:109817192 | YES | 1:109817192 | 1:109817590 | 1:109818530 | 1:109818306 |  |
| 1:115753482 | NO | 1:115771616 | 1:115777538 | 1:115723695 | 1:115759830 |  |
| 1:151762308 | NO | 1:151735576 | 1:151744928 | 1:151728756 |  |  |
| 1:154395946 | YES | 1:154395946 | 1:154422067 | 1:154407419 | 1:154418749 |  |
| 1:169094459 | NO | 1:169095312 | 1:169090660 | 1:169088132 | 1:169313682 |  |
| 1:200646073 | NO | 1:200639683 | 1:200592275 | 1:200662215 | 1:200557767 |  |
| 1:201872264 | NO | 1:201860626 | 1:201865763 | 1:201800868 | 1:201884952 |  |
| 1:210468999 | NO | 1:210467047 | 1:210443474 | 1:210510894 |  |  |
| 1:222762709 | NO | 1:222772139 | 1:222763235 | 1:222751034 | 1:222786032 |  |
| 1:222823743 | YES | 1:222823743 | 1:222814442 | 1:222823529 | 1:222826481 |  |
| 1:230845794 | YES | 1:230845794 | 1:230849359 | 1:230844310 | 1:230849799 |  |
| 2:19942473 | YES | 2:19942473 | 2:19945577 | 2:19918336 | 2:19931143 |  |
| 2:21286057 | YES | 2:21286057 | 2:21290067 | 2:21291529 | 2:21294975 |  |
| 2:44073881 | YES | 2:44073881 | 2:44074431 | 2:44072576 | 2:44098855 |  |
| 2:45896437 | NO | 2:45864710 | 2:45884565 | 2:45902454 | 2:45865779 |  |
| 2:85788175 | YES | 2:85809989 | 2:85738374 | 2:85755357 |  |  |
| 2:145286559 | YES | 2:145286559 | 2:145288341 | 2:145285067 | 2:145254129 |  |
| 2:145801461 | YES | 2:145801461 | 2:145825555 | 2:145837441 | 2:145822778 |  |
| 2:164957251 | NO | 2:164934709 | 2:165056704 | 2:164973051 | 2:164990304 |  |
| 2:188196469 | YES | 2:188196469 | 2:188333064 | 2:188343497 | 2:188083123 |  |
| 2:203745885 | YES | 2:203745885 | 2:203670122 | 2:203679306 | 2:203680954 |  |
| 2:216291359 | NO | 2:216299281 | 2:216313591 | 2:216285375 | 2:216300185 |  |
| 2:216304384 | YES | 2:216304384 | 2:216300185 | 2:216276991 | 2:216299281 |  |
| 2:218683154 | NO | 2:218695102 | 2:218680529 | 2:218694551 |  |  |
| 2:227100698 | YES | 2:227100698 | 2:227099854 | 2:227099180 | 2:227116365 |  |
| 2:233633460 | NO | 2:233699415 | 2:233723182 | 2:233592501 | 2:233699459 |  |
| 2:238223955 | NO | 2:238227919 | 2:238232752 | 2:238214039 |  |  |
| 3:14928077 | NO | 3:14958126 | 3:14928335 | 3:14939479 | 3:14932156 |  |
| 3:46688562 | NO | 3:46688498 | 3:46677872 |  |  |  |
| 3:48193515 | NO | 3:48287879 | 3:48303669 | 3:48339699 | 3:48340045 |  |
| 3:49448566 | NO | 3:49453834 | 3:49455330 | 3:49441091 | 3:49860854 |  |
| 3:124475201 | NO | 3:124478734 | 3:124585532 | 3:124577555 |  |  |
| 3:132257961 | NO | 3:132366498 | 3:132260432 | 3:132294075 | 3:132218623 |  |
| 3:136069472 | NO | 3:136255169 | 3:136069286 | 3:135926622 |  |  |
| 3:138122122 | YES | 3:138122122 | 3:138119952 | 3:138087467 |  |  |
| 3:153839866 | YES | 3:153839866 | 3:153779868 | 3:153875713 | 3:153766181 |  |
| 3:156852592 | NO | 3:156852205 | 3:156836517 | 3:156828823 | 3:156849811 |  |
| 3:172115902 | NO | 3:172119811 |  |  |  |  |
| 4:3449652 | YES | 4:3449652 | 4:3517779 |  |  |  |
| 4:57838583 | YES | 4:57838583 | 4:57823476 | 4:57857188 | 4:57733604 |  |
| 4:77416627 | NO | 4:77437749 | 4:77392092 | 4:77364799 | 4:77420784 |  |
| 4:81181072 | NO | 4:81184341 | 4:81169912 | 4:81164723 | 4:81158545 |  |
| 4:82587050 | YES | 4:82587050 | 4:82620131 | 4:82602057 |  |  |
| 4:96117371 | NO | 4:96116778 | 4:96108829 | 4:96106322 | 4:96123466 |  |
| 4:120909501 | NO | 4:120847421 | 4:120846553 | 4:120821994 | 4:120910200 |  |
| 4:148281001 | YES | 4:148281001 | 4:148288067 | 4:148289389 | 4:148291438 |  |
| 4:156639888 | YES | 4:156639888 | 4:156635309 | 4:156675593 | 4:156645513 |  |
| 4:169687725 | NO | 4:169689516 | 4:169725605 | 4:169684612 | 4:169703442 |  |
| 5:9556694 | NO | 5:9547129 | 5:9604225 | 5:9558232 | 5:9579513 |  |
| 5:55860781 | NO | 5:55859952 | 5:55860866 | 5:55861786 | 5:55861894 |  |
| 5:121413208 | YES | 5:121413208 | 5:121422057 | 5:121419259 |  |  |
| 5:131667353 | NO | 5:131785536 | 5:131649186 | 5:131793772 |  |  |
| 5:142516897 | YES | 5:142516897 | 5:142507651 | 5:142516670 | 5:142519870 |  |
| 6:1617143 | NO | 6:1613686 |  |  |  |  |
| 6:2933355 | NO | 6:2937347 |  |  |  |  |
| 6:11327021 | YES | 6:11327021 | 6:11321650 | 6:11266238 | 6:11305230 |  |
| 6:11774583 | YES | 6:11774583 |  |  |  |  |
| 6:12903957 | YES | 6:12903957 | 6:12907591 | 6:12909874 | 6:12889004 |  |
| 6:22612629 | NO | 6:22571037 | 6:22504878 | 6:22545166 | 6:22600813 |  |
| 6:31888367 | YES | 6:31888367 | 6:31845985 | 6:31812038 | 6:32050544 |  |
| 6:34769765 | NO | 6:34728253 | 6:34763982 | 6:34683635 |  |  |
| 6:35034800 | YES | 6:35034800 | 6:34898455 | 6:35049999 |  |  |
| 6:36638636 | YES | 6:36638636 | 6:36632688 | 6:36629444 | 6:36645588 |  |
| 6:39134099 | YES | 6:39134099 | 6:39136651 | 6:39149784 | 6:39157781 |  |
| 6:43758873 | YES | 6:43758873 | 6:43757896 | 6:43764551 |  |  |
| 6:57160572 | NO | 6:57126898 | 6:57144935 | 6:57128259 | 6:57112128 |  |
| 6:82612271 | NO | 6:82611058 | 6:82599523 | 6:82617837 |  |  |
| 6:126717064 | NO | 6:126759033 | 6:126767600 | 6:126698719 | 6:126781434 |  |
| 6:134173151 | NO | 6:134196674 | 6:134209837 | 6:134151297 | 6:134123832 |  |
| 6:150997401 | NO | 6:151004770 | 6:150997440 | 6:150975312 |  |  |
| 6:160863532 | YES | 6:160863532 | 6:160872151 | 6:160858188 | 6:160840252 |  |
| 6:160961137 | YES | 6:160961137 | 6:160922870 |  |  |  |
| 6:161005610 | NO | 6:161010118 | 6:161108144 | 6:160906086 | 6:160907134 |  |
| 6:161143608 | YES | 6:161143608 | 6:161143376 | 6:161152240 | 6:161152294 |  |
| 7:1937261 | NO | 7:1950809 | 7:1892309 | 7:1969345 | 7:1920826 |  |
| 7:12261911 | NO | 7:12265988 | 7:12283787 | 7:12227023 | 7:12227892 |  |
| 7:19049388 | NO | 7:19037661 | 7:19035920 | 7:19036775 | 7:19031935 |  |
| 7:45077978 | NO | 7:45192586 |  |  |  |  |
| 7:107176780 | NO | 7:107096144 | 7:107283069 | 7:107066030 | 7:107029879 |  |
| 7:107244545 | YES | 7:107244545 | 7:107207695 | 7:107113850 | 7:107096144 |  |
| 7:129663496 | YES | 7:129663496 | 7:129653231 |  |  |  |
| 7:139757136 | YES | 7:139757136 | 7:139723400 | 7:139726207 | 7:139719211 |  |
| 7:150690176 | YES | 7:150690176 | 7:150684021 | 7:150673314 | 7:150689943 |  |
| 8:18286997 | NO | 8:18288772 | 8:18262861 | 8:18259366 | 8:18272881 |  |
| 8:19813180 | YES | 8:19813180 | 8:19808030 | 8:19815189 | 8:19865843 |  |
| 8:22033615 | NO | 8:22037736 |  |  |  |  |
| 8:106565414 | NO | 8:106581284 | 8:106593558 | 8:106573578 | 8:106556634 |  |
| 8:126490972 | YES | 8:126485337 | 8:126479315 | 8:126479362 |  |  |
| 9:22003790 | NO | 9:22115105 | 9:21936381 | 9:22074793 |  |  |
| 9:22125503 | YES | 9:22124630 | 9:22124477 |  |  |  |
| 9:110517794 | NO | 9:110522741 | 9:110536932 | 9:110514284 |  |  |
| 9:113169775 | YES | 9:113169775 | 9:113140259 | 9:113153329 | 9:113196733 |  |
| 9:124420173 | NO | 9:124422403 | 9:124412961 | 9:124419014 |  |  |
| 9:136141870 | NO | 9:136149399 | 9:136155000 | 9:136153875 | 9:136154168 |  |
| 10:12303813 | NO | 10:12288302 | 10:12277992 | 10:12253597 | 10:12249797 |  |
| 10:30323892 | YES | 10:30323892 | 10:30306804 | 10:30335122 |  |  |
| 10:44480811 | YES | 10:44480811 | 10:44494546 | 10:44500807 | 10:44515716 |  |
| 10:44753867 | YES | 10:44753867 | 10:44751910 | 10:44752330 | 10:44752976 |  |
| 10:82251514 | NO | 10:82262826 | 10:82238403 | 10:82277341 | 10:82223767 |  |
| 10:91002927 | YES | 10:91002927 | 10:91005854 | 10:91003419 | 10:91004886 |  |
| 10:104604916 | YES | 10:104604916 | 10:104591393 | 10:104594507 |  |  |
| 10:105693644 | NO | 10:105720022 | 10:105702042 | 10:105683494 | 10:105672842 |  |
| 10:124237612 | NO | 10:124237338 | 10:124259062 | 10:124277713 |  |  |
| 11:9751196 | NO | 11:9800601 | 11:9784616 | 11:9791579 |  |  |
| 11:10284499 | NO | 11:10212021 | 11:10184205 | 11:10034540 | 11:10019879 |  |
| 11:10745394 | YES | 11:10745394 | 11:10737001 | 11:10715124 |  |  |
| 11:13303071 | NO | 11:13312969 | 11:13315439 | 11:13267554 | 11:13350747 |  |
| 11:43696917 | NO | 11:43648368 | 11:43839935 | 11:43877934 |  |  |
| 11:65349063 | NO | 11:65349756 | 11:65353906 | 11:65363958 | 11:65368323 |  |
| 11:65391317 | YES | 11:65391317 | 11:65408937 | 11:65531222 | 11:65460957 |  |
| 11:75274150 | NO | 11:75283653 | 11:75279476 | 11:75275004 |  |  |
| 11:100624599 | NO | 11:100633303 | 11:100638603 | 11:100639302 | 11:100582941 |  |
| 11:103673277 | YES | 11:103673277 | 11:103660567 | 11:103674339 | 11:103703939 |  |
| 11:116648917 | YES | 11:116648917 | 11:116657561 | 11:116647607 | 11:116653296 |  |
| 12:7175872 | NO | 12:7170336 | 12:7165114 | 12:7156051 | 12:7176618 |  |
| 12:20220033 | NO | 12:20237578 | 12:20217448 | 12:20214966 | 12:20218803 |  |
| 12:57527283 | YES | 12:57527283 | 12:57515363 | 12:57531632 | 12:57534912 |  |
| 12:57843711 | NO | 12:57844049 | 12:57809456 | 12:57792580 | 12:57789359 |  |
| 12:90008959 | YES | 12:90008959 | 12:90026523 | 12:90060586 | 12:89942390 |  |
| 12:95355541 | YES | 12:95355541 | 12:95442118 | 12:95544908 | 12:95345522 |  |
| 12:111884608 | YES | 12:111884608 | 12:111910219 | 12:112007756 |  |  |
| 12:118265441 | NO | 12:118265149 | 12:118272871 | 12:118279944 | 12:118286251 |  |
| 12:121416988 | YES | 12:121416988 | 12:121416650 | 12:121420807 |  |  |
| 12:124427306 | NO | 12:124440110 | 12:124486678 | 12:124499839 | 12:124400261 |  |
| 12:125307053 | YES | 12:125307053 | 12:125316743 | 12:125296964 |  |  |
| 13:28973621 | YES | 13:28973621 | 13:29022645 | 13:28966067 |  |  |
| 13:33058333 | NO | 13:33184288 | 13:33147052 | 13:33147548 | 13:32998610 |  |
| 13:110818102 | YES | 13:110818102 | 13:110817641 | 13:110821084 |  |  |
| 13:110960712 | YES | 13:110960712 | 13:110957662 | 13:110955187 | 13:110954981 |  |
| 13:111040681 | YES | 13:111040681 | 13:111040798 | 13:111033821 | 13:111015780 |  |
| 13:111049623 | YES | 13:111049623 | 13:111033821 | 13:111054682 | 13:111047350 |  |
| 13:113631780 | NO | 13:113659108 | 13:113657867 | 13:113633796 |  |  |
| 14:58794001 | NO | 14:58821929 | 14:58831142 | 14:58653514 | 14:58713559 |  |
| 14:94838142 | YES | 14:94838142 | 14:94844947 | 14:94672731 | 14:94863636 |  |
| 14:100145710 | NO | 14:100133942 | 14:100133250 | 14:100111955 |  |  |
| 15:65024204 | NO | 15:64965815 | 15:64672159 | 15:64524980 |  |  |
| 15:67455630 | YES | 15:67455630 | 15:67442596 | 15:67450305 | 15:67468285 |  |
| 15:79124475 | NO | 15:79111093 | 15:79121373 | 15:79117133 | 15:79118296 |  |
| 15:89574218 | NO | 15:89566607 | 15:89581128 | 15:89564843 | 15:89561223 |  |
| 15:91416550 | YES | 15:91416550 | 15:91429042 | 15:91426560 |  |  |
| 15:96146414 | NO | 15:96167827 | 15:96179023 | 15:96145098 |  |  |
| 16:56995236 | YES | 16:56995236 | 16:56996288 | 16:56996649 | 16:56996211 |  |
| 16:72130815 | NO | 16:72050480 | 16:72042682 | 16:72108093 | 16:72230112 |  |
| 16:75387533 | NO | 16:75331044 | 16:75313258 | 16:75328308 |  |  |
| 16:81906423 | NO | 16:81905504 | 16:81900628 | 16:81909265 | 16:81899211 |  |
| 16:83045790 | NO | 16:82999663 | 16:83039645 | 16:83042938 |  |  |
| 17:2126504 | YES | 17:2125605 | 17:2117945 | 17:2143460 |  |  |
| 17:17543722 | YES | 17:17543722 | 17:17544704 | 17:17538008 | 17:17612023 |  |
| 17:27941886 | NO | 17:27935688 | 17:28349095 | 17:27959903 | 17:27924179 |  |
| 17:30033514 | NO | 17:30033834 | 17:30021321 | 17:30022353 | 17:30018832 |  |
| 17:40257163 | YES | 17:40257163 | 17:40289364 | 17:40305542 | 17:40257055 |  |
| 17:45013271 | YES | 17:45013271 | 17:45054564 | 17:45073761 | 17:45008570 |  |
| 17:46988597 | YES | 17:46988597 | 17:46974734 | 17:46988529 | 17:46987665 |  |
| 17:47123423 | YES | 17:47123423 | 17:47106878 | 17:47067826 |  |  |
| 17:59013488 | NO | 17:59017025 | 17:59022395 | 17:58916059 | 17:59161646 |  |
| 17:62387091 | NO | 17:62401118 | 17:62387211 | 17:62408299 | 17:62354992 |  |
| 18:47229717 | NO | 18:47250428 | 18:47268505 | 18:47230431 | 18:47205432 |  |
| 18:57838401 | NO | 18:57829135 | 18:57839769 | 18:57849023 |  |  |
| 19:8429323 | YES | 19:8429323 |  |  |  |  |
| 19:11163601 | YES | 19:11163601 | 19:11171600 | 19:11166293 |  |  |
| 19:11188247 | YES | 19:11188247 | 19:11188117 | 19:11188153 | 19:11188164 |  |
| 19:17855763 | NO | 19:17872069 | 19:17859370 | 19:17829608 | 19:17834759 |  |
| 19:32882020 | YES | 19:32882020 | 19:32970870 | 19:32835191 | 19:32828993 |  |
| 19:33386556 | NO | 19:33372814 | 19:33358527 | 19:33467413 | 19:33466932 |  |
| 19:41854534 | NO | 19:41851509 | 19:41860296 | 19:41883198 |  |  |
| 19:45422946 | NO | 19:45422846 | 19:45410002 | 19:45414451 |  |  |
| 19:46190268 | NO | 19:46208023 | 19:46191828 | 19:46185217 |  |  |
| 20:33764554 | YES | 20:33764554 | 20:33768523 | 20:33769926 | 20:33775200 |  |
| 20:39924279 | NO | 20:39928646 | 20:39698734 | 20:39841091 |  |  |
| 20:44586023 | NO | 20:44538427 | 20:44594228 | 20:44540178 | 20:44542832 |  |
| 20:57714025 | NO | 20:57705485 | 20:57691416 | 20:57745815 | 20:57751117 |  |
| 21:30533076 | NO | 21:30567941 | 21:30546000 | 21:30524293 | 21:30518636 |  |
| 21:35593827 | YES | 21:35593827 | 21:35599128 | 21:35637462 | 21:35655777 |  |
| 22:24658858 | NO | 22:24679907 | 22:24744702 | 22:24745877 | 22:24888192 |  |

| **Supplementary Table 3** proxy lookup, further details. This table is for information only, where palindromic SNPs/proxies are not removed. Palindromic SNPs are highlighted in yellow; palindromic SNPs with RAF between 0.35 and 0.65 are in red; For genotyped published SNPs, only the palindromic SNPs with RAF between 0.35 and 0.65 are recommended to be replaced | | | | | | |
| --- | --- | --- | --- | --- | --- | --- |
| 3SNPs from literature | genotyped | selected | proxy1 | proxy2 | proxy3 | comment |
| 1:2252205 | NO | 1:2251160 | 1:2225284 | #NV | #NV |  |
| 1:3325912 | NO | 1:3328659 | 1:3313282 | 1:3308498 | #NV |  |
| 1:38461319 | NO | 1:38456106 | 1:38453576 | 1:38449910 | 1:38401933 |  |
| 1:55496039 | YES | 1:55496039 | 1:55487648 | #NV | #NV |  |
| 1:55505647 | YES | 1:55505647 | #NV | #NV | #NV |  |
| 1:56965664 | YES | 1:56965664 | 1:56966350 | 1:56962821 | 1:56986978 |  |
| 1:109817192 | YES | 1:109817192 | 1:109817590 | 1:109818530 | 1:109818306 |  |
| 1:115753482 | NO | 1:115771616 | 1:115777538 | 1:115723695 | 1:115759830 |  |
| 1:151762308 | NO | 1:151735576 | 1:151744928 | 1:151728756 | #NV |  |
| 1:154395946 | YES | 1:154395946 | 1:154422067 | 1:154407419 | 1:154418749 |  |
| 1:169094459 | NO | 1:169095312 | 1:169090660 | 1:169088132 | 1:169313682 |  |
| 1:200646073 | NO | 1:200639683 | 1:200592275 | 1:200662215 | 1:200557767 |  |
| 1:201872264 | NO | 1:201860626 | 1:201865763 | 1:201800868 | 1:201884952 |  |
| 1:210468999 | NO | 1:210467047 | 1:210443474 | 1:210510894 | #NV |  |
| 1:222762709 | NO | 1:222772139 | 1:222763235 | 1:222751034 | 1:222786032 |  |
| 1:222823743 | YES | 1:222823743 | 1:222814442 | 1:222823529 | 1:222826481 |  |
| 1:230845794 | YES | 1:230845794 | 1:230849359 | 1:230844310 | 1:230849799 |  |
| 2:19942473 | YES | 2:19942473 | 2:19945577 | 2:19918336 | 2:19931143 |  |
| 2:21286057 | YES | 2:21286057 | 2:21290067 | 2:21291529 | 2:21294975 |  |
| 2:44073881 | YES | 2:44073881 | 2:44074431 | 2:44072576 | 2:44098855 |  |
| 2:45896437 | NO | 2:45864710 | 2:45884565 | 2:45902454 | 2:45865779 |  |
| 2:85788175 | YES | 2:85788175 | 2:85809989 | 2:85738374 | 2:85755357 | selected: palindromic SNP with RAF between 0.35 and 0.65 |
| 2:145286559 | YES | 2:145286559 | 2:145288341 | 2:145285067 | 2:145254129 |  |
| 2:145801461 | YES | 2:145801461 | 2:145825555 | 2:145837441 | 2:145822778 |  |
| 2:164957251 | NO | 2:164934709 | 2:165056704 | 2:164973051 | 2:164990304 |  |
| 2:188196469 | YES | 2:188196469 | 2:188333064 | 2:188343497 | 2:188083123 |  |
| 2:203745885 | YES | 2:203745885 | 2:203670122 | 2:203679306 | 2:203680954 |  |
| 2:216291359 | NO | 2:216299281 | 2:216313591 | 2:216285375 | 2:216300185 |  |
| 2:216304384 | YES | 2:216304384 | 2:216300185 | 2:216276991 | 2:216299281 |  |
| 2:218683154 | NO | 2:218695102 | 2:218680529 | 2:218694551 | #NV |  |
| 2:227100698 | YES | 2:227100698 | 2:227099854 | 2:227099180 | 2:227116365 |  |
| 2:233633460 | NO | 2:233699415 | 2:233723182 | 2:233592501 | 2:233699459 |  |
| 2:238223955 | NO | 2:238227919 | 2:238232752 | 2:238214039 | #NV |  |
| 3:14928077 | NO | 3:14958126 | 3:14928335 | 3:14939479 | 3:14932156 |  |
| 3:46688562 | NO | 3:46688498 | 3:46677872 | #NV | #NV |  |
| 3:48193515 | NO | 3:48287879 | 3:48303669 | 3:48339699 | 3:48340045 |  |
| 3:49448566 | NO | 3:49453834 | 3:49455330 | 3:49441091 | 3:49860854 |  |
| 3:124475201 | NO | 3:124478734 | 3:124585532 | 3:124577555 | #NV |  |
| 3:132257961 | NO | 3:132366498 | 3:132260432 | 3:132294075 | 3:132218623 |  |
| 3:136069472 | NO | 3:136255169 | 3:136069286 | 3:135926622 | #NV |  |
| 3:138122122 | YES | 3:138122122 | 3:138119952 | 3:138087467 | 3:138088064 | proxy3: palindromic SNP |
| 3:153839866 | YES | 3:153839866 | 3:153779868 | 3:153875713 | 3:153766181 | selected: palindromic SNP |
| 3:156852592 | NO | 3:156852205 | 3:156836517 | 3:156828823 | 3:156849811 |  |
| 3:172115902 | NO | 3:172119811 | #NV | #NV | #NV |  |
| 4:3449652 | YES | 4:3449652 | 4:3517779 | #NV | #NV |  |
| 4:57838583 | YES | 4:57838583 | 4:57823476 | 4:57857188 | 4:57733604 |  |
| 4:77416627 | NO | 4:77437749 | 4:77392092 | 4:77364799 | 4:77420784 |  |
| 4:81181072 | NO | 4:81184341 | 4:81169912 | 4:81164723 | 4:81158545 | selected: palindromic SNP |
| 4:82587050 | YES | 4:82587050 | 4:82620131 | 4:82602057 | #NV |  |
| 4:96117371 | NO | 4:96116778 | 4:96108829 | 4:96106322 | 4:96123466 |  |
| 4:120909501 | NO | 4:120847421 | 4:120846553 | 4:120821994 | 4:120910200 |  |
| 4:148281001 | YES | 4:148281001 | 4:148288067 | 4:148289389 | 4:148291438 | selected: palindromic SNP |
| 4:156639888 | YES | 4:156639888 | 4:156635309 | 4:156675593 | 4:156645513 |  |
| 4:169687725 | NO | 4:169689516 | 4:169725605 | 4:169684612 | 4:169703442 |  |
| 5:9556694 | NO | 5:9547129 | 5:9604225 | 5:9558232 | 5:9579513 |  |
| 5:55860781 | NO | 5:55859952 | 5:55860866 | 5:55861786 | 5:55861894 |  |
| 5:121413208 | YES | 5:121413208 | 5:121422057 | 5:121419259 | #NV |  |
| 5:131667353 | NO | 5:131785536 | 5:131703578 | 5:131649186 | 5:131793772 | proxy1: palindromic SNP |
| 5:142516897 | YES | 5:142516897 | 5:142507651 | 5:142516670 | 5:142519870 |  |
| 6:1617143 | NO | 6:1613686 | #NV | #NV | #NV |  |
| 6:2933355 | NO | 6:2937347 | #NV | #NV | #NV |  |
| 6:11327021 | YES | 6:11327021 | 6:11321650 | 6:11266238 | 6:11305230 |  |
| 6:11774583 | YES | 6:11774583 | #NV | #NV | #NV |  |
| 6:12903957 | YES | 6:12903957 | 6:12907591 | 6:12909874 | 6:12889004 |  |
| 6:22612629 | NO | 6:22571037 | 6:22504878 | 6:22545166 | 6:22600813 |  |
| 6:31888367 | YES | 6:31888367 | 6:31845985 | 6:31812038 | 6:32050544 |  |
| 6:34769765 | NO | 6:34728253 | 6:34763982 | 6:34683635 | #NV |  |
| 6:35034800 | YES | 6:35034800 | 6:34898455 | 6:35049999 | #NV | selected: palindromic SNP |
| 6:36638636 | YES | 6:36638636 | 6:36632688 | 6:36629444 | 6:36645588 |  |
| 6:39134099 | YES | 6:39134099 | 6:39136651 | 6:39149784 | 6:39157781 |  |
| 6:43758873 | YES | 6:43758873 | 6:43757896 | 6:43764551 | #NV |  |
| 6:57160572 | NO | 6:57126898 | 6:57144935 | 6:57128259 | 6:57112128 |  |
| 6:82612271 | NO | 6:82611058 | 6:82599523 | 6:82617837 | #NV |  |
| 6:126717064 | NO | 6:126759033 | 6:126767600 | 6:126698719 | 6:126781434 |  |
| 6:134173151 | NO | 6:134196674 | 6:134209837 | 6:134151297 | 6:134123832 |  |
| 6:150997401 | NO | 6:151004770 | 6:150997440 | 6:150975312 | #NV | selected: palindromic SNP |
| 6:160863532 | YES | 6:160863532 | 6:160872151 | 6:160858188 | 6:160840252 |  |
| 6:160961137 | YES | 6:160961137 | 6:160922870 | #NV | #NV |  |
| 6:161005610 | NO | 6:161010118 | 6:161108144 | 6:160906086 | 6:160907134 |  |
| 6:161143608 | YES | 6:161143608 | 6:161143376 | 6:161152240 | 6:161152294 |  |
| 7:1937261 | NO | 7:1950809 | 7:1892309 | 7:1969345 | 7:1920826 |  |
| 7:12261911 | NO | 7:12265988 | 7:12283787 | 7:12227023 | 7:12227892 |  |
| 7:19049388 | NO | 7:19037661 | 7:19035920 | 7:19036775 | 7:19031935 |  |
| 7:45077978 | NO | 7:45192586 | #NV | #NV | #NV |  |
| 7:107176780 | NO | 7:107096144 | 7:107283069 | 7:107066030 | 7:107029879 |  |
| 7:107244545 | YES | 7:107244545 | 7:107207695 | 7:107113850 | 7:107096144 |  |
| 7:129663496 | YES | 7:129663496 | 7:129653231 | #NV | #NV |  |
| 7:139757136 | YES | 7:139757136 | 7:139723400 | 7:139726207 | 7:139719211 |  |
| 7:150690176 | YES | 7:150690176 | 7:150684021 | 7:150673314 | 7:150689943 |  |
| 8:18286997 | NO | 8:18288772 | 8:18262861 | 8:18259366 | 8:18272881 |  |
| 8:19813180 | YES | 8:19813180 | 8:19808030 | 8:19815189 | 8:19865843 |  |
| 8:22033615 | NO | 8:22037736 | #NV | #NV | #NV |  |
| 8:106565414 | NO | 8:106581284 | 8:106593558 | 8:106573578 | 8:106556634 |  |
| 8:126490972 | YES | 8:126490972 | 8:126485337 | 8:126479315 | 8:126479362 | selected: palindromic SNP with RAF between 0.35 and 0.65 |
| 9:22003790 | NO | 9:22115105 | 9:21936381 | 9:22074793 | #NV |  |
| 9:22125503 | YES | 9:22125503 | 9:22124630 | 9:22124477 | 9:22124744 | selected and proxy3: palindromic SNP with RAF between 0.35 and 0.65 |
| 9:110517794 | NO | 9:110522741 | 9:110536932 | 9:110514284 | #NV |  |
| 9:113169775 | YES | 9:113169775 | 9:113140259 | 9:113153329 | 9:113196733 |  |
| 9:124420173 | NO | 9:124422403 | 9:124412961 | 9:124419014 | #NV |  |
| 9:136141870 | NO | 9:136149399 | 9:136155000 | 9:136153875 | 9:136154168 |  |
| 10:12303813 | NO | 10:12288302 | 10:12277992 | 10:12253597 | 10:12249797 |  |
| 10:30323892 | YES | 10:30323892 | 10:30306804 | 10:30316072 | 10:30335122 | proxy2: palindromic SNP with RAF between 0.35 and 0.65 |
| 10:44480811 | YES | 10:44480811 | 10:44494546 | 10:44500807 | 10:44515716 |  |
| 10:44753867 | YES | 10:44753867 | 10:44751910 | 10:44752330 | 10:44752976 |  |
| 10:82251514 | NO | 10:82262826 | 10:82238403 | 10:82277341 | 10:82223767 |  |
| 10:91002927 | YES | 10:91002927 | 10:91005854 | 10:91003419 | 10:91004886 |  |
| 10:104604916 | YES | 10:104604916 | 10:104591393 | 10:104594507 | 10:104595849 | proxy3: palindromic SNP |
| 10:105693644 | NO | 10:105720022 | 10:105702042 | 10:105683494 | 10:105672842 |  |
| 10:124237612 | NO | 10:124237338 | 10:124259062 | 10:124277713 | #NV |  |
| 11:9751196 | NO | 11:9800601 | 11:9784616 | 11:9791579 | #NV |  |
| 11:10284499 | NO | 11:10212021 | 11:10184205 | 11:10034540 | 11:10019879 |  |
| 11:10745394 | YES | 11:10745394 | 11:10740366 | 11:10737001 | 11:10715124 | proxy1: palindromic SNP with RAF between 0.35 and 0.65 |
| 11:13303071 | NO | 11:13312969 | 11:13315439 | 11:13267554 | 11:13350747 |  |
| 11:43696917 | NO | 11:43648368 | 11:43839935 | 11:43877934 | #NV |  |
| 11:65349063 | NO | 11:65349756 | 11:65353906 | 11:65363958 | 11:65368323 |  |
| 11:65391317 | YES | 11:65391317 | 11:65408937 | 11:65531222 | 11:65460957 |  |
| 11:75274150 | NO | 11:75283653 | 11:75279476 | 11:75275004 | #NV |  |
| 11:100624599 | NO | 11:100633303 | 11:100638603 | 11:100639302 | 11:100582941 |  |
| 11:103673277 | YES | 11:103673277 | 11:103660567 | 11:103674339 | 11:103703939 |  |
| 11:116648917 | YES | 11:116648917 | 11:116657561 | 11:116647607 | 11:116653296 | selected: palindromic SNP |
| 12:7175872 | NO | 12:7170336 | 12:7165114 | 12:7156051 | 12:7176618 |  |
| 12:20220033 | NO | 12:20237578 | 12:20217448 | 12:20214966 | 12:20218803 |  |
| 12:57527283 | YES | 12:57527283 | 12:57515363 | 12:57531632 | 12:57534912 |  |
| 12:57843711 | NO | 12:57844049 | 12:57809456 | 12:57792580 | 12:57789359 |  |
| 12:90008959 | YES | 12:90008959 | 12:90026523 | 12:90060586 | 12:89942390 |  |
| 12:95355541 | YES | 12:95355541 | 12:95442118 | 12:95544908 | 12:95345522 |  |
| 12:111884608 | YES | 12:111884608 | 12:111904371 | 12:111910219 | 12:112007756 | proxy1: palindromic SNP with RAF between 0.35 and 0.65 |
| 12:118265441 | NO | 12:118265149 | 12:118272871 | 12:118279944 | 12:118286251 |  |
| 12:121416988 | YES | 12:121416988 | 12:121416650 | 12:121420807 | #NV |  |
| 12:124427306 | NO | 12:124440110 | 12:124486678 | 12:124499839 | 12:124400261 |  |
| 12:125307053 | YES | 12:125307053 | 12:125312425 | 12:125316743 | 12:125296964 | proxy1: palindromic SNP |
| 13:28973621 | YES | 13:28973621 | 13:28984063 | 13:29022645 | 13:28966067 | proxy1: palindromic SNP |
| 13:33058333 | NO | 13:33184288 | 13:33147052 | 13:33147548 | 13:32998610 |  |
| 13:110818102 | YES | 13:110818102 | 13:110817641 | 13:110821084 | #NV | selected: palindromic SNP |
| 13:110960712 | YES | 13:110960712 | 13:110957662 | 13:110955187 | 13:110954981 |  |
| 13:111040681 | YES | 13:111040681 | 13:111040798 | 13:111033821 | 13:111015780 |  |
| 13:111049623 | YES | 13:111049623 | 13:111033821 | 13:111054682 | 13:111047350 |  |
| 13:113631780 | NO | 13:113659108 | 13:113657867 | 13:113633796 | #NV |  |
| 14:58794001 | NO | 14:58821929 | 14:58831142 | 14:58653514 | 14:58713559 |  |
| 14:94838142 | YES | 14:94838142 | 14:94844947 | 14:94672731 | 14:94863636 |  |
| 14:100145710 | NO | 14:100133942 | 14:100127440 | 14:100133250 | 14:100111955 | proxy1: palindromic SNP with RAF between 0.35 and 0.65 |
| 15:65024204 | NO | 15:64965815 | 15:64672159 | 15:64524980 | #NV |  |
| 15:67455630 | YES | 15:67455630 | 15:67442596 | 15:67450305 | 15:67468285 |  |
| 15:79124475 | NO | 15:79111093 | 15:79121373 | 15:79117133 | 15:79118296 |  |
| 15:89574218 | NO | 15:89566607 | 15:89581128 | 15:89564843 | 15:89561223 |  |
| 15:91416550 | YES | 15:91416550 | 15:91429042 | 15:91426560 | 15:91420940 | proxy3: palindromic SNP |
| 15:96146414 | NO | 15:96167827 | 15:96179023 | 15:96145098 | #NV |  |
| 16:56995236 | YES | 16:56995236 | 16:56996288 | 16:56996649 | 16:56996211 |  |
| 16:72130815 | NO | 16:72050480 | 16:72042682 | 16:72108093 | 16:72230112 |  |
| 16:75387533 | NO | 16:75331044 | 16:75313258 | 16:75328308 | 16:75450362 | proxy3: the same location has an indel and a palindromic SNP |
| 16:81906423 | NO | 16:81905504 | 16:81900628 | 16:81909265 | 16:81899211 |  |
| 16:83045790 | NO | 16:82999663 | 16:83039645 | 16:83042938 | 16:83060151 | proxy3: palindromic SNP |
| 17:2126504 | YES | 17:2126504 | 17:2125605 | 17:2117945 | 17:2143460 | selected: palindromic SNP with RAF between 0.35 and 0.65 |
| 17:17543722 | YES | 17:17543722 | 17:17544704 | 17:17538008 | 17:17612023 |  |
| 17:27941886 | NO | 17:27935688 | 17:28349095 | 17:27959903 | 17:27924179 |  |
| 17:30033514 | NO | 17:30033834 | 17:30021321 | 17:30022353 | 17:30018832 |  |
| 17:40257163 | YES | 17:40257163 | 17:40289364 | 17:40305542 | 17:40257055 |  |
| 17:45013271 | YES | 17:45013271 | 17:45054564 | 17:45073761 | 17:45008570 |  |
| 17:46988597 | YES | 17:46988597 | 17:46974734 | 17:46988529 | 17:46987665 |  |
| 17:47123423 | YES | 17:47123423 | 17:47106878 | 17:47067826 | #NV |  |
| 17:59013488 | NO | 17:59017025 | 17:59022395 | 17:58916059 | 17:59161646 |  |
| 17:62387091 | NO | 17:62401118 | 17:62387211 | 17:62408299 | 17:62354992 |  |
| 18:47229717 | NO | 18:47250428 | 18:47268505 | 18:47230431 | 18:47205432 |  |
| 18:57838401 | NO | 18:57829135 | 18:57839769 | 18:57848369 | 18:57849023 | proxy2: palindromic SNP |
| 19:8429323 | YES | 19:8429323 | #NV | #NV | #NV |  |
| 19:11163601 | YES | 19:11163601 | 19:11179709 | 19:11171600 | 19:11166293 | proxy1: palindromic SNP |
| 19:11188247 | YES | 19:11188247 | 19:11188117 | 19:11188153 | 19:11188164 |  |
| 19:17855763 | NO | 19:17872069 | 19:17859370 | 19:17829608 | 19:17834759 |  |
| 19:32882020 | YES | 19:32882020 | 19:32970870 | 19:32835191 | 19:32828993 | selected: palindromic SNP |
| 19:33386556 | NO | 19:33372814 | 19:33358527 | 19:33467413 | 19:33466932 |  |
| 19:41854534 | NO | 19:41851509 | 19:41860296 | 19:41883198 | #NV |  |
| 19:45422946 | NO | 19:45422846 | 19:45410002 | 19:45414451 | #NV |  |
| 19:46190268 | NO | 19:46208023 | 19:46191828 | 19:46185217 | #NV |  |
| 20:33764554 | YES | 20:33764554 | 20:33768523 | 20:33769926 | 20:33775200 |  |
| 20:39924279 | NO | 20:39928646 | 20:39698734 | 20:39672618 | 20:39841091 | proxy2: palindromic SNP with RAF between 0.35 and 0.65 |
| 20:44586023 | NO | 20:44538427 | 20:44594228 | 20:44540178 | 20:44542832 |  |
| 20:57714025 | NO | 20:57705485 | 20:57691416 | 20:57745815 | 20:57751117 |  |
| 21:30533076 | NO | 21:30567941 | 21:30546000 | 21:30524293 | 21:30518636 |  |
| 21:35593827 | YES | 21:35593827 | 21:35599128 | 21:35637462 | 21:35655777 |  |
| 22:24658858 | NO | 22:24679907 | 22:24744702 | 22:24745877 | 22:24888192 |  |

**Supplementary Table 4** **Studies used to identify CAD-associated SNPs.**

| Reference number | Reference | Comments |
| --- | --- | --- |
| ^1^ | Roberts 2018 | These were the most recent and thorough reports on CAD genetics at the time our studies began. |
| ^2^ | van der Harst and Verweij 2018 |  |
| ^3^ | Nikpav et al. 2015 | These 3 reports confirmed SNPs found in Roberts (2018) and van der Harst and Verweij (2018). |
| ^4^ | Howson et al. 2017 |  |
| ^5^ | Klarin et al. 2017 |  |
| ^6^ | Schunkert et al. 2011 | These were state of the art papers based on large consortia. |
| ^7^ | Consortium CAD et al. 2013 |  |
| ^8^ | Helgadottir et al. 2007 | These reports contained additional SNPs specific to 9p21 and provided further proxy SNPs for this region. |
| ^9^ | Kral et al. 2011 |  |
| ^10^ | Nelson et al. 2017 | These papers contained additional SNPs not reported in Roberts (2018) and van der Harst and Verweij (2018). |
| ^11^ | Reilly et al. 2011 |  |
| ^12^ | Consortium IKC 2011 |  |

**Harmonization of SNP effects.** We performed several harmonization steps before constructing the PRS. Specifically,

1. Gathering SNP information:
   - From each paper, we extracted: SNP IDs (e.g., rsID); effect alleles (alleles associated with increased risk); effect sizes [β, log(OR), or OR]; and P-values.
   - We converted all SNP coordinates to the same genome build using LiftOver.
2. Harmonizing alleles for each SNP:

- We aligned effect alleles.
- We flipped alleles if necessary.
- We identified potential replacements for **palindromic SNPs** (A/T or C/G), especially for SNPs with an allele frequency >0.35.

1. Handling duplicate or overlapping SNPs:

Only one core SNP in each LD block was kept in the PRS calculation, to avoid double counting.

- We used the following procedure for SNPs that appeared in multiple studies:
  - The priority was to keep SNPs from the paper with PubMed ID 29319564. If a SNP in the LD block was in that paper, then we picked that SNP as the core SNP in the region.
  - If none of the SNPs in the LD block was in paper 29319564, then the core SNP was the SNP that was in more papers than the other SNPs in the LD block.
  - If all SNPs in the LD block were represented equally among the papers, then we used the SNP from the study with the largest sample size.
- Different SNPs may be in linkage disequilibrium (LD). Hence, we applied LD clumping to remove correlated SNPs with a threshold of R^2^>0.2.

We removed 76 SNPs from the original set of 259 by LD pruning (r^2^<0.2). Additionally, we removed 2 SNPs with no sufficient proxies, leading to the final set of 181 SNPs for PRS construction.

1. Weighting strategy

We calculated each PRS using the weight for each SNP divided by the mean weight for all SNPs, i.e.
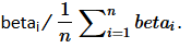


In this way, each PRS is based on the relative contribution of each SNP to the overall genetic risk for CAD.

1. Standardizing the score: After computing the PRS, we normalized it to mean = 0 and SD = 1, to make interpretation easier across datasets.
2. Validating the harmonization procedure: We compared our PRS constructed from the 181 SNPs from multiple papers to a PRS constructed from 46 SNPs reported in Hajek et al.^13^. The 46 SNPs were identified in a single study (**Supplementary Table 5**).

**Supplementary Table 5** **Validation using 181 vs 46 SNPs for CAD PRS in MESA sample for incident CHD**

Comparison of the 181-SNP CAD PRS to the 46-SNP CAD PRS in relation to incident CAD in The Multi-Ethnic Study of Atherosclerosis (MESA). The tables below show associates between incident CHD and GRS quartile for males and females separately using a Cox proportional hazard model, adjusting for are and BMI. 1A displays results for the 181-SNP PRS. 1B displays results for the 46-SNP PRS.

| **GRS Quartile** | **1 (reference)** | **2** | **3** | **4** | ***p*-value for trend** |
| --- | --- | --- | --- | --- | --- |
| **A.** | | | | | |
| Hazard Ratio (95% CI) for incident CHD vs reference | | | | | |
| **Females** | 1 | 1.104  (0.5985-2.037) | 1.149  (0.6307-2.092) | 1.132  (0.6174-2.075) | 0.1484 |
| **Males** | 1 | 1.212  (0.7158-2.053) | 1.775  (1.0785-2.921) | 2.195  (1.3506-3.569) | 0.0131 |
| **B.** | | | | | |
| Hazard Ratio (95% CI) for incident CHD vs reference | | | | | |
| **Females** | 1 | 1.282  (0.733-2.242) | 0.831  (0.453-1.524) | 0.725  (0.380-1.381) | 0.3223 |
| **Males** | 1 | 1.204  (0.7356-1.972) | 1.371  (0.837-2.245) | 1.742  (1.090-2.785) | 0.0168 |

**References:**

1. Roberts R. Genetics in the prevention and management of coronary artery disease. *Curr Opin Cardiol*. 2018;33:257-268. doi: 10.1097/HCO.0000000000000501

2. van der Harst P, Verweij N. Identification of 64 Novel Genetic Loci Provides an Expanded View on the Genetic Architecture of Coronary Artery Disease. *Circ Res*. 2018;122:433-443. doi: 10.1161/CIRCRESAHA.117.312086

3. Nikpay M, Goel A, Won HH, Hall LM, Willenborg C, Kanoni S, Saleheen D, Kyriakou T, Nelson CP, Hopewell JC, et al. A comprehensive 1,000 Genomes-based genome-wide association meta-analysis of coronary artery disease. *Nat Genet*. 2015;47:1121-1130. doi: 10.1038/ng.3396

4. Howson JMM, Zhao W, Barnes DR, Ho WK, Young R, Paul DS, Waite LL, Freitag DF, Fauman EB, Salfati EL, et al. Fifteen new risk loci for coronary artery disease highlight arterial-wall-specific mechanisms. *Nat Genet*. 2017;49:1113-1119. doi: 10.1038/ng.3874

5. Klarin D, Zhu QM, Emdin CA, Chaffin M, Horner S, McMillan BJ, Leed A, Weale ME, Spencer CCA, Aguet F, et al. Genetic analysis in UK Biobank links insulin resistance and transendothelial migration pathways to coronary artery disease. *Nat Genet*. 2017;49:1392-1397. doi: 10.1038/ng.3914

6. Schunkert H, Konig IR, Kathiresan S, Reilly MP, Assimes TL, Holm H, Preuss M, Stewart AF, Barbalic M, Gieger C, et al. Large-scale association analysis identifies 13 new susceptibility loci for coronary artery disease. *Nat Genet*. 2011;43:333-338. doi: 10.1038/ng.784

7. Consortium CAD, Deloukas P, Kanoni S, Willenborg C, Farrall M, Assimes TL, Thompson JR, Ingelsson E, Saleheen D, Erdmann J, et al. Large-scale association analysis identifies new risk loci for coronary artery disease. *Nat Genet*. 2013;45:25-33. doi: 10.1038/ng.2480

8. Helgadottir A, Thorleifsson G, Manolescu A, Gretarsdottir S, Blondal T, Jonasdottir A, Jonasdottir A, Sigurdsson A, Baker A, Palsson A, et al. A common variant on chromosome 9p21 affects the risk of myocardial infarction. *Science*. 2007;316:1491-1493. doi: 10.1126/science.1142842

9. Kral BG, Mathias RA, Suktitipat B, Ruczinski I, Vaidya D, Yanek LR, Quyyumi AA, Patel RS, Zafari AM, Vaccarino V, et al. A common variant in the CDKN2B gene on chromosome 9p21 protects against coronary artery disease in Americans of African ancestry. *J Hum Genet*. 2011;56:224-229. doi: 10.1038/jhg.2010.171

10. Nelson CP, Goel A, Butterworth AS, Kanoni S, Webb TR, Marouli E, Zeng L, Ntalla I, Lai FY, Hopewell JC, et al. Association analyses based on false discovery rate implicate new loci for coronary artery disease. *Nat Genet*. 2017;49:1385-1391. doi: 10.1038/ng.3913

11. Reilly MP, Li M, He J, Ferguson JF, Stylianou IM, Mehta NN, Burnett MS, Devaney JM, Knouff CW, Thompson JR, et al. Identification of ADAMTS7 as a novel locus for coronary atherosclerosis and association of ABO with myocardial infarction in the presence of coronary atherosclerosis: two genome-wide association studies. *Lancet*. 2011;377:383-392. doi: 10.1016/S0140-6736(10)61996-4

12. Consortium IKC. Large-scale gene-centric analysis identifies novel variants for coronary artery disease. *PLoS Genet*. 2011;7:e1002260. doi: 10.1371/journal.pgen.1002260

13. Hajek C, Guo X, Yao J, Hai Y, Johnson WC, Frazier-Wood AC, Post WS, Psaty BM, Taylor KD, Rotter JI. Coronary Heart Disease Genetic Risk Score Predicts Cardiovascular Disease Risk in Men, Not Women. *Circ Genom Precis Med*. 2018;11:e002324. doi: 10.1161/CIRCGEN.118.002324
